# T2T Genome Assembly and Multi‐Omics Data Reveal Terrestrial Adaptation and Mucus Biosynthesis in Tropical Leatherleaf Slug (*Laevicaulis alte*)

**DOI:** 10.1002/advs.76129

**Published:** 2026-06-15

**Authors:** Gang Wang, Shaofang He, Zhongkai Wang, Aobo Pang, Xiaoli Sun, Rongchen Liu, Fang Wang, Sisi Chen, Zhijuan Bian, Dongcheng Wei, Liuhan Wu, Siyao Zhao, Qiuting Ji, Yiqi Sun, Niying Sun, Yushinta Fujaya, Boping Tang, Kianann Tan, Daizhen Zhang, Lianfu Chen

**Affiliations:** ^1^ Jiangsu Provincial Key Laboratory of Coastal Wetland Bioresources and Environmental Protection Yancheng Teachers University Yancheng China; ^2^ College of Plant Science and Technology Huazhong Agricultural University Wuhan China; ^3^ Wuhan Carboncode Biotechnologies Co., Ltd. Wuhan China; ^4^ New Cornerstone Science Laboratory Northwestern Polytechnical University Xi'an China; ^5^ Guangxi Key Lab for Sugarcane Biology Guangxi University Nanning China; ^6^ Faculty of Marine Science and Fisheries Hasanuddin University Makassar Indonesia; ^7^ Guangxi Key Laboratory of Beibu Gulf Marine Biodiversity Conservation Beibu Gulf University Qinzhou China

**Keywords:** adaptive evolution, gap‐free genome, *Laevichaulis alte*, VEGF

## Abstract

*Laevichaulis alte* is a slug in the order Systellommatophora that evolved from aquatic ancestors and now faces strong challenges from desiccation, respiration on land, and novel pathogens. Its mucus is essential for water retention, locomotion, and defense. To link terrestrial adaptation with mucus biosynthesis, we generated a gap‐free genome assembly of *L. alte* using PacBio HiFi reads, Oxford Nanopore ultra‐long reads, and Hi‐C data. The genome shows low heterozygosity and holocentromeric chromosomes. Functional metabolomics revealed marked metabolic shifts between *L. alte* and the closely related aquatic species *Peronia verruculata*. In *L. alte*, differential metabolites were enriched in lipid metabolism, immune regulation, and stress response pathways, consistent with life in a dry and microbe‐rich terrestrial environment. Comparative genomics and transcriptomics identified candidate genes linked to mucus secretion and physiological adaptation, including *VEGF*, *ASGR2*, and *COL6A6*. Further analyses highlighted the vascular endothelial growth factor (VEGF) gene family as a key regulator connecting angiogenesis, tissue remodeling, and mucus production pathways in *L. alte*. Together, this gap‐free genome and multi‐omics dataset establish a molecular framework that links genomic innovation, mucus biology, and terrestrial adaptation in Systellommatophora, and they offer a basis for understanding ecological niche specialization in land molluscs.

## Graphical abstract

A gap‐free genome assembly and multi‐omics comparison of the terrestrial slug *Laevichaulis alte* with an aquatic relative reveal that expansion of the VEGF family orchestrates mucus production, lipid metabolism, and immune defense—highlighting key molecular innovations for conquering life on land.

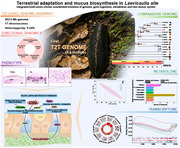

## 1 Introduction

Systellommatophora (Mollusca: Gastropoda) includes both aquatic or semi‐terrestrial onchidiids and fully terrestrial veronicellids. This clade is therefore a useful system for studying the ecological and genomic basis of the transition from water to land [1, 2]. Onchidiids occupy intertidal habitats and still rely on aquatic or humid environments, whereas veronicellids complete their life cycle on land and must cope with air breathing, desiccation, and new communities of pathogens and symbionts [1, 2]. These differences within a single order provide a natural framework to compare the genetic changes that underlie terrestrial adaptation.

For soft‐bodied slugs, mucus secretion is central to this transition. Veronicellids depend on large amounts of mucus to prevent water loss, support locomotion on abrasive surfaces, and form a physical and chemical barrier against predators and pathogens [3, 4, 5]. Mucus is produced by specialized gland cells in the epidermis and underlying tissues and is essential for water retention, protection, and movement [6]. Its adhesive and elastic properties also help deter predators, which often cannot overcome its stickiness [6]. Previous study shows that slug mucus is mainly composed of water, glycoproteins, mucopolysaccharides, and other macromolecules that form a hydrated gel with adjustable viscosity [7]. The composition and cross‐linking of this gel can change rapidly, which allows slugs to adjust viscosity and adhesion in response to environmental and behavioral demands. Mucus production is thought to be tightly regulated, involving complex signaling pathways that control secretion, glycosylation, and chemical modification of its components [8, 9]. However, the molecular mechanisms that link gene regulation, metabolism, and mucus biosynthesis remain poorly understood. Clarifying these mechanisms is important not only for understanding terrestrial adaptation in molluscs, but also for potential applications in biomimetic materials and biomedicine, where slug mucus has been explored as a natural bio‐adhesive in surgical and tissue‐engineering contexts [10, 11].

Genomic resources are essential to dissect these mechanisms. However, high‐quality genomes of terrestrial molluscs are still rare [12, 13]. The first reported genome of a land snail, *Achatina fulica*, provided initial insight into gene family dynamics linked to terrestrial life [14]. Later, a high‐quality genome of *A. immaculata* highlighted the impact of whole‐genome duplication on adaptive evolution [15]. A chromosome‐level slug genome of the *Arion vulgaris* [16], and more recent draft genomic data for *Meghimatium bilineatum* [17], have been released. However, the genome of the leatherleaf slugs (Veronicellidae) within the order Systellommatophora remains unreported, and Mollusca currently lack gap‐free genomes. We still lack a complete chromosomal framework to study structural variation, repetitive elements, and gene family evolution in this group. This gap in genomic resources also constrains efforts to link genotype, mucus phenotype, and terrestrial adaptation.

*Laevicaulis alte*, commonly known as the tropical leatherleaf slug or black slug, is a veronicellid species widely distributed in tropical regions [18]. It has expanded from its native range into many parts of southern and southeastern Asia, where it feeds on a broad spectrum of crops and ornamental plants [19, 20]. Its high reproductive capacity, generalist feeding behavior, and ability to thrive in disturbed habitats make *L. alte* a serious agricultural pest that can cause substantial damage to crop and horticultural systems [21, 22]. At the same time, *L. alte* functions as a decomposer that contributes to nutrient cycling by breaking down plant litter [23]. The species therefore has both economic and ecological importance. Its heavy reliance on mucus for locomotion, feeding, and defense, together with its broad environmental tolerance, makes *L. alte* an informative model for studying the molecular basis of terrestrial adaptation and mucus biology in Veronicellidae.

Here, we present a gap‐free, telomere‐to‐telomere (T2T) genome assembly of *L. alte* and integrate it with transcriptomic and metabolomic data. The complete genome provides a high‐resolution view of chromosome organization, centromere structure, repetitive landscapes, and gene family architecture in a terrestrial slug. By combining this resource with tissue‐specific transcriptomes and metabolomics, we investigate the pathways that underpin mucus biosynthesis and secretion. This integrated multi‐omics framework links genomic innovation to physiological function and ecological performance in *L. alte*. It advances our understanding of terrestrial adaptation in Systellommatophora, informs the development of more targeted management strategies for this pest species, and provides a molecular basis for the biomimetic use of gastropod mucus in materials science and biomedicine.

## 2 Results

### 2.1 A High‐quality Low‐Heterozygosity Genome Assembly and Annotation for *L. alte*

**FIGURE 1:**
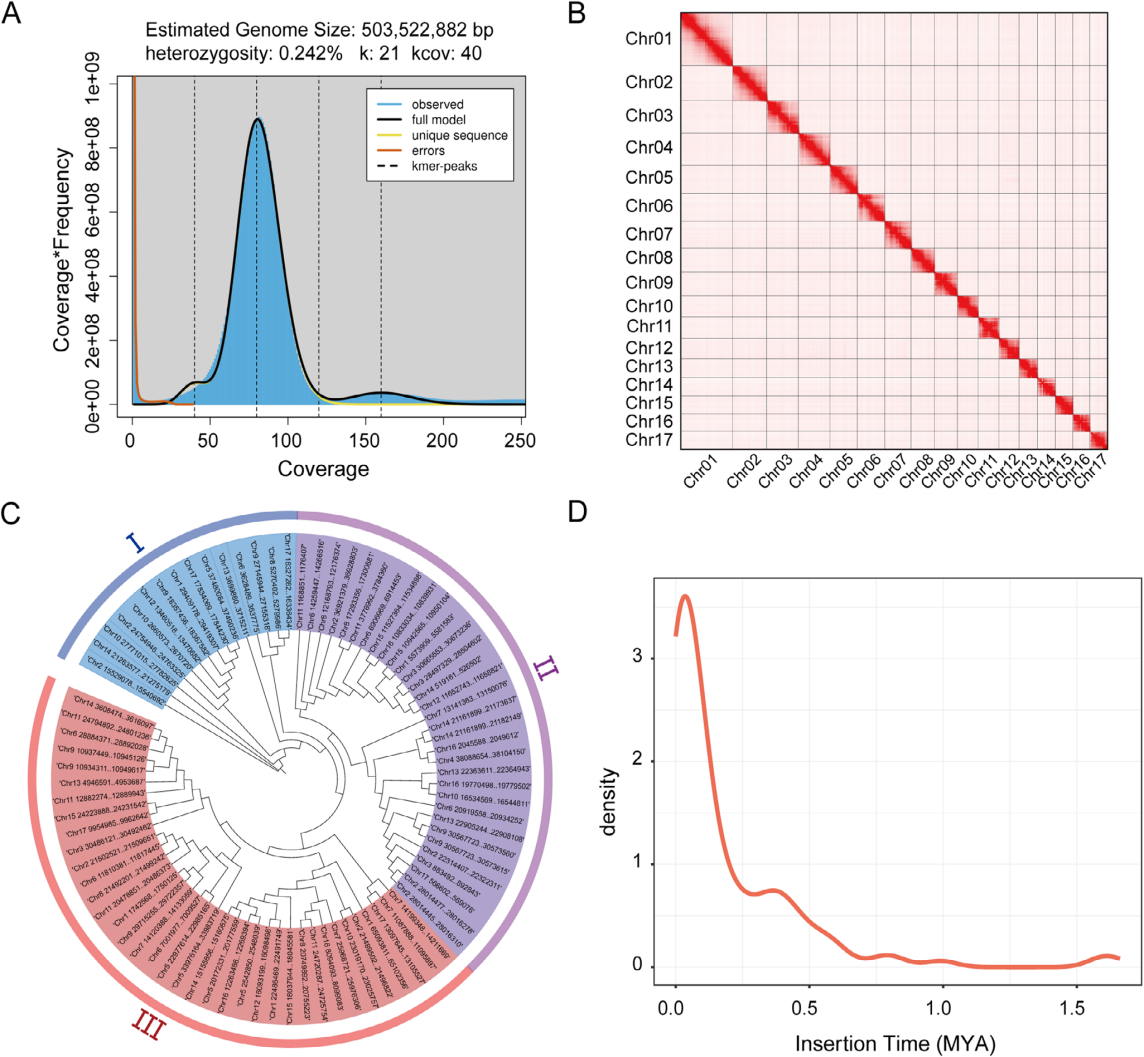
Telomere‐to‐telomere‐level assembly reveals a distinctive *L. alte* genome. (A) K‐mer distribution and genome size estimation. (B) Chromosome Hi‐C interaction heat map displays the physical interaction frequencies between chromosomal regions as measured. (C) The circular phylogenetic tree of gene or repeat families. (D) The transposable element insertion time plot.

A genome survey based on 56 Gb (100×) of Illumina short reads estimated *L. alte* genome size at 503.52 Mb and revealed very low heterozygosity (0.24%), consistent with an inbred or low‐diversity lineage (Figure 1A and Table S1). To obtain a high‐quality assembly suitable for downstream multi‐omics and adaptation analyses, we combined 29.2 Gb of PacBio HiFi reads (≈58.4×), 29.5 Gb of ONT ultra‐long reads (≈59×), and 91 Gb of Hi‐C data (≈156×) (Table S2; Figures S1 and S2). De novo assembly with HiFi and ultra‐long reads followed by Hi‐C scaffolding allowed us to anchor 45 contigs onto 17 chromosomes with an anchoring rate of 99.28% (Figure 1B and Table S3).

To close residual gaps, we extracted 10 kb flanking sequences on both sides of each gap and aligned them to draft genome sequences used for gap filling. Before gap closure, the 17 chromosomes contained 27 gaps and lacked two telomeres (the 3′ telomere of Chr1 and the 5′ telomere of Chr14) (Table S4). After iterative gap filling, all chromosomes carried complete telomeres, yielding 34 telomeres in total and achieving a T2T assembly. The final T2T assembly size is 586.03 Mb, and the contig N50 increased from 21.03 to 36.85 Mb (Table S5 and Figure S3). Assembly completeness was high, with BUSCO scores of 96.75% (metazoa_odb10) and 95.33% (mollusca_odb10) (Figure S4), indicating that the *L. alte* genome provides a robust foundation for exploring terrestrial adaptation and mucus‐related gene families.

A Circos plot summarizes the chromosomal landscape and highlights the uneven distribution of repeats, genes, and other genomic features across the 17 chromosomes (Figure S5). A circular phylogram further resolves sequence relationships into three major clades (I, II, III), labeled in blue, purple, and red (Figure 1C). Closely clustered tips indicate recent duplication events, whereas deeper branches point to older divergence, suggesting a layered history of expansion and diversification in key genomic regions.

Repetitive elements account for 22.99% of the T2T genome, including 10.23% retrotransposons and 1.64% DNA transposons. We identified 300 complete LTR elements, of which 229 belong to the Bel‐Pao subclass (Table S6). The distribution of transposable element (TE) insertion times shows a clear peak around 0.1 million years ago (MYA) (Figure 1D). This recent burst‐like TE activity likely contributed to genome expansion in *L. alte*.

Gene annotation based on protein homology and RNA‐seq evidence identified 24,193 high‐confidence protein‐coding genes after removal of transposon‐related genes. BUSCO analysis of the predicted gene set showed high completeness, with scores of 99.2% (metazoa_odb10) and 95.3% (mollusca_odb10) (Figure S6). This comprehensive, gap‐free gene catalog forms the basis for subsequent multi‐omics analyses of mucus biosynthesis, immunity, and stress responses that underpin terrestrial adaptation in *L. alte*.

### 2.2 Centromere Identification Suggests Holocentric Chromosomes in *L. alte*

Most chromosomes showed entropy values close to 1.0 (Figure S7), which indicates an almost even use of A, C, G, and T along large parts of the genome. Such high‐entropy regions are often associated with gene‐rich or functionally diverse regions and are easier to resolve in a gap‐free, T2T assembly. In contrast, each chromosome also contained clear entropy dips below this baseline. These low‐entropy regions likely correspond to repetitive or compositionally biased DNA, such as homopolymer tracts, GC‐ or AT‐rich runs, or microsatellite expansions. In these domains, a limited set of nucleotides dominates, which reduces sequence complexity.

The depth and frequency of these entropy troughs differed among chromosomes, suggesting variation in the size and distribution of repetitive blocks or transposon‐rich segments. In the integrated chromosome plots, entropy troughs often aligned with features in the associated “linguistic complexity” and histogram tracks, which likely mark repeat density or other structural features, whereas putative gene‐rich regions tended to retain high entropy. These patterns point to a genome‐wide balance between forces that promote uniform base composition and local processes that drive sequence repetition and bias, such as transposon bursts or tandem repeat expansion.

Taken together, the smooth, longitudinal distribution of high‐entropy regions along whole chromosomes, interspersed with localized low‐complexity blocks, is consistent with a holocentric chromosome organization in *L. alte*. This pattern supports the view that centromeric activity is distributed along the chromosome rather than confined to a single, discrete primary constriction, and it provides a structural context for later analyses of gene families and regulatory regions involved in terrestrial adaptation and mucus biosynthesis.

### 2.3 Time‐Calibrated Phylogenetic Reconstruction and Gene‐Family Dynamics for *L. alte*

**FIGURE 2:**
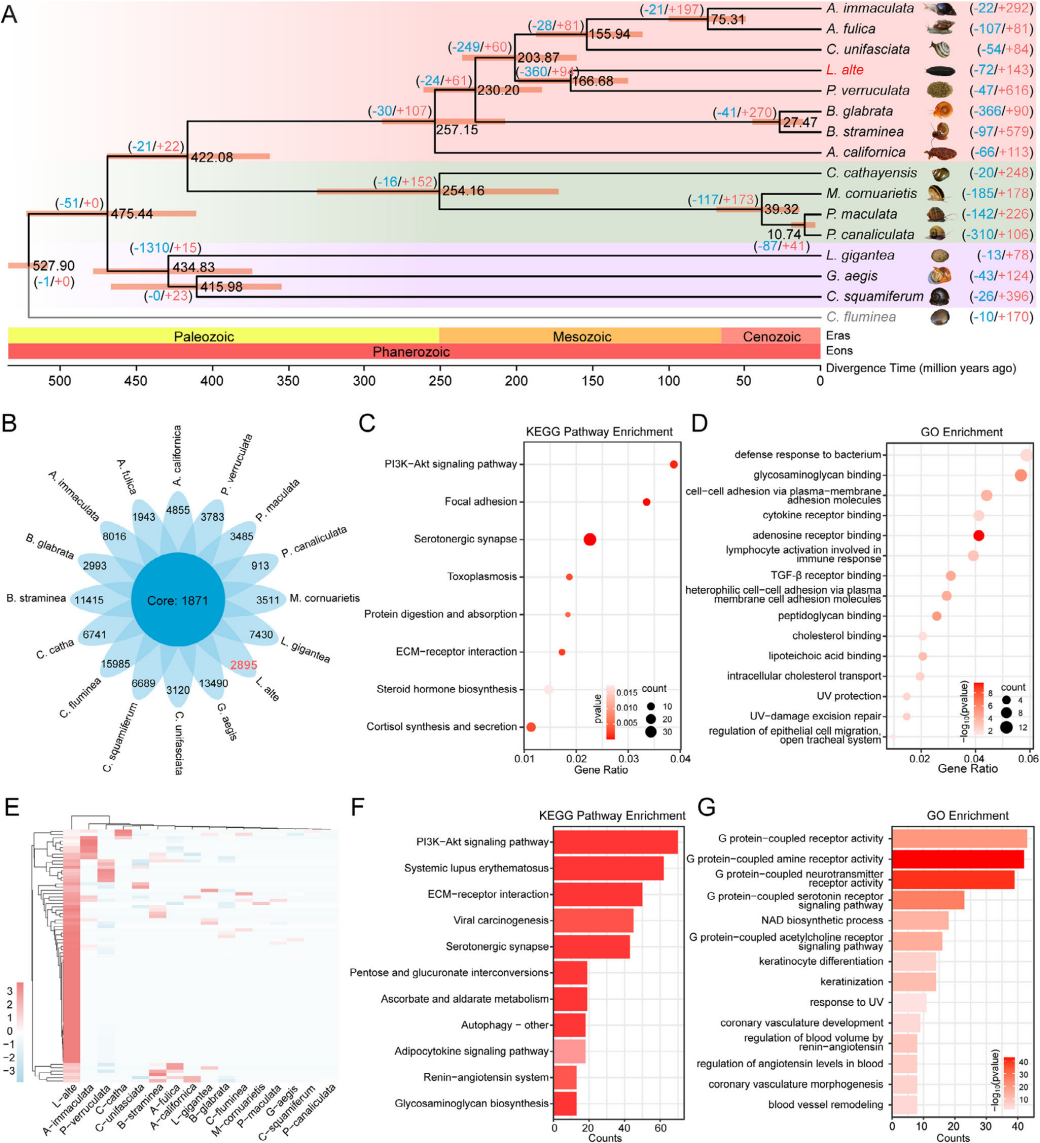
The time‐calibrated phylogenetic reconstruction and gene family dynamics for *L. alte*. (A) Phylogenetic tree of 15 gastropods with bivalve outgroup (*Corbicula fluminea*). Nodes show divergence times with 95% HPD intervals (red bars). Numbers in parentheses indicate significantly expanded (red) and contracted (blue) gene families inferred by CAFE. Full species names and accession numbers are listed in Table S7. (B) Petal diagram of core orthogroups (OGs) and species‑specific gene counts. The center number indicates the number of OGs that are shared by all species. The number on each petal represents the total number of genes from that species that fall into species‑specific OGs. (C) KEGG and (D) GO enrichment of *L. alte* species‑specific genes. (E) Heatmap of gene counts for 143 significantly expanded gene families along the *L. alte* branch, together with the corresponding gene counts for orthologous families in the other gastropod species examined. (F) KEGG and (G) GO enrichment of the 143 significantly expanded gene families.

With the genomic architecture established, we next investigated evolutionary relationships and gene‐family dynamics to identify candidate adaptive features. The time‐calibrated phylogeny clarifies the evolutionary position of *L. alte* among gastropods (Figure 2A and Table S7). *L. alte* forms a clade with *Peronia verruculata*, another member of the order Systellommatophora. This clade subsequently groups with other terrestrial molluscs, including *A. immaculata*, *A. fulica*, and *Candidula unifasciata*, forming a broader terrestrial clade. Although *P. verruculata* is regarded as an amphibious transitional species, it has evolved air‐breathing organs, supporting its affinity with terrestrial lineages. Divergence time estimates suggest that the *L. alte* and *P. verruculata* lineages separated before the Cenozoic, indicating an extended evolutionary history of adaptation to land environments.

Orthologous gene cluster analysis identified a core set of 1,871 gene families shared across the surveyed gastropod species. In contrast, *L. alte* harbors 2,895 species‐specific genes that form uniquely expanded gene families (Figure 2B). KEGG enrichment analysis of these expanded genes identified significant enrichment in pathways that are linked to cell adhesion and extracellular matrix organization, including the PI3K−Akt signaling pathway, focal adhesion, ECM–receptor interaction, and serotonergic synapse (Figure 2C). The results of the GO enrichment analysis indicate that these genes are closely associated with defense responses to bacteria, glycosaminoglycan binding, peptidoglycan binding, cholesterol binding, and UV protection (Figure 2D). These results indicate functional enrichment in pathways and biological processes related to cellular interaction and response mechanisms. These enriched functions, particularly those related to extracellular matrix organization, defense response, and environmental protection, guided our subsequent focus on mucus‐associated traits.

We further identified 143 significantly expanded gene families along the *L. alte* branch, as inferred by CAFE (Figure 2A), and subsequently examined whether these families exhibit similar patterns in other gastropods (Figure 2E). Most of these orthogroups were expanded specifically in *L. alte*. Functional enrichment analysis showed significant enrichment in pathways including the PI3K‐Akt signaling pathway, ECM‐receptor interaction, and serotonergic synapse (Figure 2F). In addition, GO enrichment analysis identified overrepresentation of terms related to G protein‐coupled receptor activity (Figure 2G), as well as terms associated with circulatory system processes, including coronary vasculature development, blood vessel remodeling, and regulation of blood volume. These enriched terms characterize the major functional features associated with the expanded gene families in *L. alte*. Because these functions are related to extracellular interactions, signaling, and physiological regulation, they provided candidate genomic features for the following functional analyses of mucus‐related adaptation. These genomic features provided a basis for subsequent metabolomic analysis of *L. alte*, aimed at characterizing biochemical changes associated with the identified gene family expansions.

### 2.4 Functional Metabolomics Identifies Key Adaptive Pathways in *L. alte*

**FIGURE 3:**
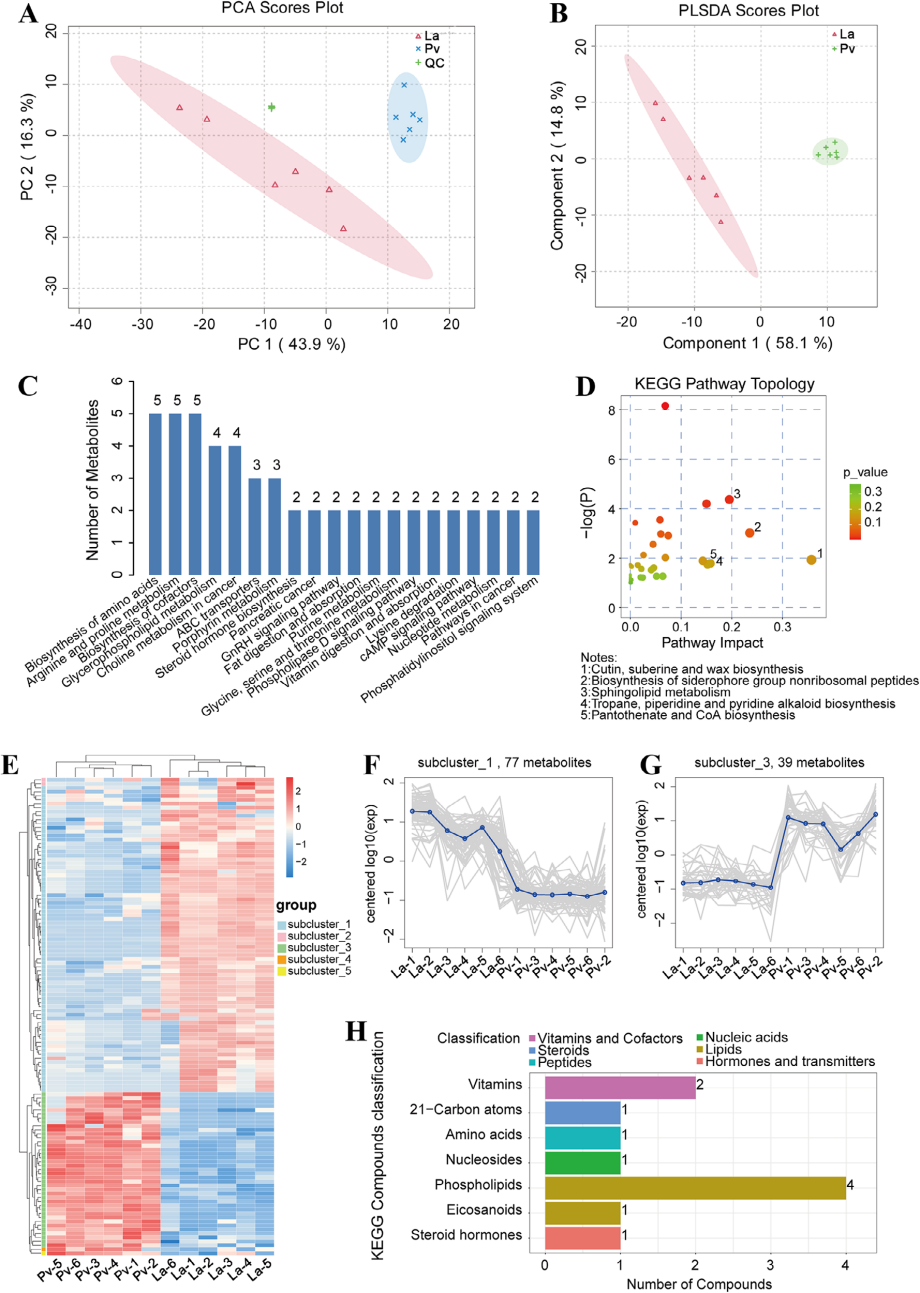
The functional metabolomics profiling of *L. alte* (La) and *P. verruculata* (Pv). (A) PCA scores plot for La and Pv samples. (B) PLSDA scores plot for La and Pv samples. (C) The bar chart of metabolites number identified in each metabolic pathway. (D) The KEGG bubble plot highlights key metabolic differences between La and Pv. (E) The metabolite clustering heat map for La and Pv. (F) The identified subcluster 1 metabolites trend for both La and Pv. (G) The identified subcluster 3 metabolites trend for both La and Pv. (H) The KEGG compounds classification chart for compounds number vs classification.

To functionally validate the genomic and evolutionary signals identified above, we next performed comparative metabolomics focusing on mucus, a key adaptive trait in *L. alte*. The markedly higher mucus production in *L. alte* compared with its close relative *P. verruculata* suggests that these sister taxa have evolved distinct strategies for environmental adaptation. To clarify the biochemical basis of this divergence, we performed comparative metabolomic profiling of mucus secretions from *L. alte* (La) and *P. verruculata* (Pv). Biological replicates within each group showed high consistency, and clear separation between groups (Figure 3A,B), indicating robust data quality and stable metabolic differences between the two species.

Across all samples, we detected 379 metabolites, of which 120 differed significantly between La and Pv; among these, 79 were upregulated and 41 were downregulated in La relative to Pv. Enriched pathways included biosynthesis of amino acids, arginine and proline metabolism, and cofactor biosynthesis (Figure 3C). The KEGG bubble plot (Figure 3D) highlights several key pathways that differ between the two mucus profiles and likely reflect adaptation to distinct ecological niches. Five pathways stood out: (i) cutin, suberine, and wax biosynthesis, which is linked to water‐loss prevention, surface protection, and reduced microbial invasion; (ii) biosynthesis of siderophore group nonribosomal peptides, associated with iron acquisition and competitive defense; (iii) sphingolipid metabolism, which affects membrane structure, signaling, and stress response; (iv) tropane, piperidine, and pyridine alkaloid biosynthesis, related to chemical defense in terrestrial biotic interactions; and (v) pantothenate and CoA biosynthesis, which underpins energy metabolism and broad physiological regulation. Together, these pathway shifts suggest that *L. alte* mucus is enriched in metabolites that support barrier function, immunity, and stress tolerance in a fully terrestrial habitat.

Hierarchical clustering further separated La from Pv (Figure 3E). Metabolites in subcluster 1 (77 metabolites), showed higher abundance in La, especially those associated with immune‐related functions/pathways (Figure 3F). These included glutamine and related compounds with known immunomodulatory effects, prostaglandins involved in inflammation and immune regulation, phenolic compounds with potential immunomodulatory roles, and peptide compounds that may participate in immune cell signaling. These features point to a mucus matrix in *L. alte* that is chemically optimized for immune defense and inflammatory control on land. In contrast, metabolites in subcluster 3 (39 metabolites) showed lower abundance in La and higher abundance in Pv (Figure 3G). Metabolites in this subcluster were relatively more abundant in Pv than in La. These metabolites are enriched in pathways associated with membrane composition, amino acid metabolism, and neuroactive signaling. These clustering patterns reflect distinct metabolite profiles between the two species.

KEGG compound classification also identified four phospholipids and two vitamins, pantothenic acid and niacinamide, as key differential metabolites (Figure 3H and Table S8). These compounds represent key categories among the significantly changed metabolites. Because several differential metabolites were associated with stress response and mucus‐related functions, we further examined transcriptional responses under salinity stress.

### 2.5 Transcriptomic Profiling and Functional Enrichment of *L. alte* Under Salinity Stress

**FIGURE 4:**
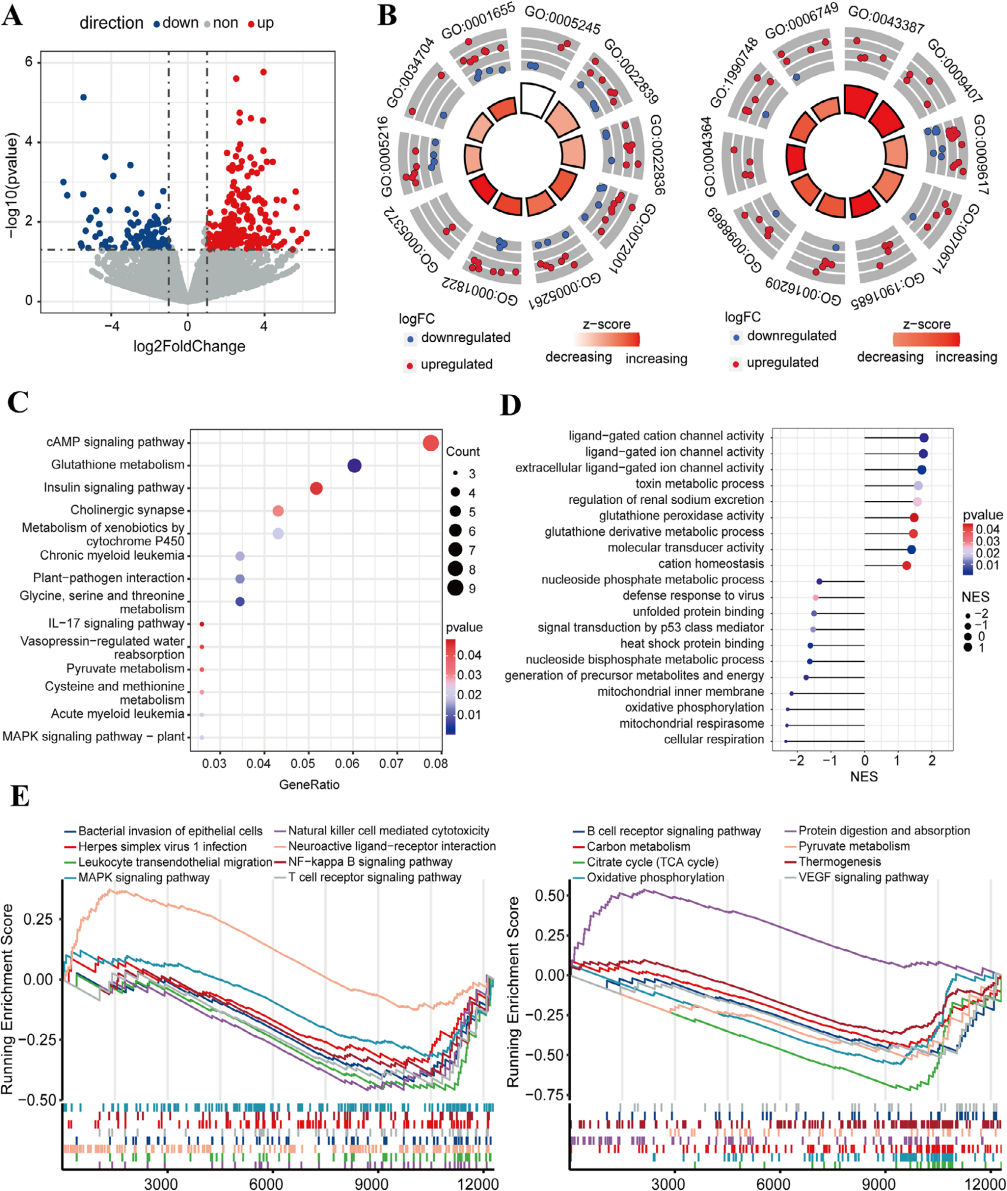
The transcriptome analysis of *L. alte* under salinity stress. (A) The volcano plot for up‐regulated and down‐regulated DEGs after salt‐treatment. (B) GO enrichment analysis of DEGs. (C) KEGG enrichment analysis of DEGs. (D) The GO terms with the top 20 |NES| obtained from GSEA in Gzy vs Gzck. (E) GSEA plots display KEGG pathways significant enriched in Gzy vs Gzck.

Building on the metabolomic evidence of adaptive biochemical pathways, we next examined transcriptional regulation to understand how these pathways are dynamically controlled under environmental stress. Because salinity exposure imposes osmotic stress and stimulates protective mucus responses in terrestrial slugs, transcriptomic profiling of *L. alte* under salt treatment was used to investigate the molecular regulation of mucus‐mediated adaptation. The gene expression profile of *L. alte* under salt stress helps clarify how mucus secretion is regulated to cope with environmental challenges. We generated high‐quality RNA‐seq data from both the control group (Gzck) and the salt‐treated group (Gzy) (Table S9). Differential expression analysis identified 340 DEGs between the two groups, including 228 upregulated and 112 downregulated genes (Figure 4A).

GO enrichment showed many terms related to immunity, such as toxin catabolic process, response to interleukin‐12, and glutathione metabolism. Mucus‐related functions were also prominent, including glutathione peroxidase activity and oxidoreductase activity acting on peroxides, which support antioxidant defense in mucus‐producing tissues. In addition, several terms were linked to vascular function and ion balance, such as voltage‐gated calcium channel activity, cation channel activity, and calcium ion binding (Figure 4B). These functions are consistent with active ion transport and fluid regulation during mucus secretion under osmotic stress.

KEGG pathway enrichment indicated that most DEGs were involved in metabolic processes, including cysteine and methionine metabolism, pyruvate metabolism, glycine/serine/threonine metabolism, xenobiotic metabolism by cytochrome P450, glutathione metabolism, and insulin signaling (Figure 4C). Some pathways were directly related to mucus secretion and epithelial regulation, such as insulin and cAMP signaling, while others were associated with water and ion handling and respiratory function, including vasopressin‐regulated water reabsorption, cholinergic synapse, cAMP signaling, and IL‐17 signaling. These results indicate that salinity treatment was accompanied by transcriptional changes in pathways related to epithelial function, ion transport, metabolism, and stress response.

We next performed gene set enrichment analysis (GSEA). The normalized enrichment score (NES) highlighted ligand‐gated cation channel activity, extracellular ligand‐gated ion channel activity, and cation homeostasis (Figure 4D). These terms are involved in ion transport, membrane excitability, and intracellular ion balance, which are essential for maintaining cell volume and mucus hydration under salinity stress. Positively enriched terms also included nucleoside phosphate metabolism and molecular transducer activity, indicating active cell signaling and high energy turnover. In contrast, oxidative phosphorylation and related mitochondrial processes showed negative NES values, suggesting a shift away from high‐rate aerobic ATP production toward protective, detoxifying, or stress‐responsive pathways.

GSEA further revealed that, under salinity stress, *L. alte* upregulated pathways such as neuroactive ligand–receptor interaction and protein digestion and absorption. This pattern suggests that *L. alte* may use neuropeptides or hormone‐like signals to adjust fluid balance, promote ion excretion, or enhance water retention when exposed to salt. Several inflammation‐ and immune‐related pathways, including NF‐κB signaling, natural killer cell–mediated cytotoxicity, T cell receptor signaling, and B cell receptor signaling, were significantly downregulated (Figure 3E). In addition, VEGF signaling, oxidative phosphorylation, and the TCA cycle were suppressed. These changes indicate a trade‐off during acute salinity stress, in which *L. alte* transiently reduces immune activation, tissue repair, and energy synthesis pathways while prioritizing ion homeostasis, mucus secretion, and immediate stress protection. These transcriptomic results highlighted stress‐responsive pathways related to ion homeostasis, neuroactive signaling, metabolism, and epithelial or mucus‐associated functions in *L. alte*, which were further examined in subsequent tissue‐level analyses.

### 2.6 Validation of Key Mucous‐Producing Genes in *L. alte* Skin Tissue Using FISH and qPCR

**FIGURE 5:**
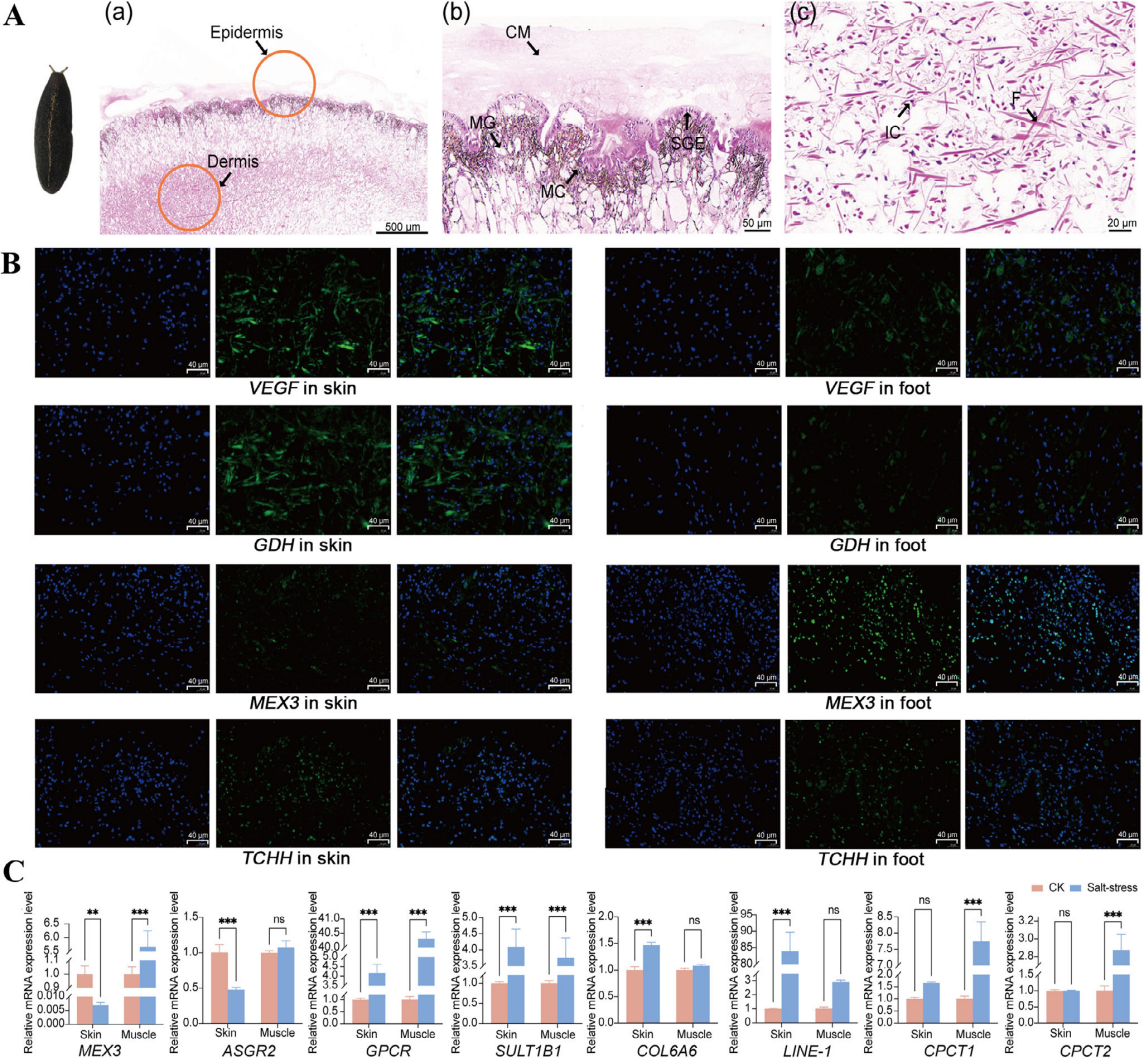
The histological observation, gene localization, distribution, and transcription in *L. alte*. (A) H&E‐stained sections of dorsal skin tissue from *L. alte*. (a) 4×. (b) 20×, The epidermis in the dorsal skin of *L. alte*; MG, mucous gland; CM, cuticular membrane; SGE, stratum germinative; MC, melanin cell. (c) 40×, The dermis in the dorsal skin of *L. alte*; F, fibers; IC, immune cell. (B) Localization and distribution of *VEGF*, *GDH*, *MEX3*, and *TCHH* in the skin and foot of *L. alte*. (C) The relative mRNA level of *MEX3*, *ASGR2*, *GPCR*, *SULT1B1*, *COL6A6*, *LINE‐1*, *CPCT1*, *CPCT2* in skin and muscle tissues under salt‐stress.

To assess tissue‐level patterns of candidate genes and pathways identified from the multi‐omics analyses, we performed histological analysis, fluorescence in situ hybridization, and qPCR‐based expression analysis. Histological analysis of H&E‐stained skin sections of *L. alte* (Figure 5A) revealed clear structural differences compared with our previous observations in *Onchidium reevesii* (Figure S8), a semi‐terrestrial gastropod with similar mucus‐secreting traits and related taxonomy. Both species share a comparable basic skin architecture. However, *L. alte* shows a much thicker cuticular membrane and a higher density of mucous glands in the epidermis. In addition, the epidermis of *L. alte* contains abundant melanocytes. These features suggest stronger protection against water loss, mechanical damage, and UV exposure, and they are consistent with a fully terrestrial lifestyle that relies heavily on a robust mucus barrier and pigmented skin for environmental defense.

Based on the multi‐omics results and tissue morphology, four representative genes were selected for spatial expression analysis: *VEGF*, *GDH*, *MEX3*, and *TCHH*. These genes represented candidate vascular, metabolic, RNA‐regulatory, and epidermal structural modules, respectively. FISH analysis revealed the spatial distribution of *VEGF*, *GDH*, *MEX3*, and *TCHH* in the skin and foot tissues of L. alte (Figure 5B). Notably, *VEGF* showed a strong fluorescence signal in the skin, consistent with its role as a representative marker of the vascular adaptation module, which is associated with angiogenesis and tissue oxygen supply. The foot also showed *VEGF* expression, but with a weaker signal than in the skin. The *GDH* signal was moderate in the skin and slightly weaker in the foot, indicating tissue‐level expression of this metabolic candidate gene*. MEX3* displayed weak, localized expression in the skin but a stronger signal in the foot, suggesting a role in RNA metabolism and cell differentiation in locomotory tissues. *TCHH* produced diffuse, moderate fluorescence signal in the skin and weaker signal in the foot, pointing to higher expression in the epidermis, where secreted proteases may contribute to mucus remodeling, surface defense, or digestion of external material. Together, these spatial patterns support a model in which vascular, metabolic, RNA regulatory, and protease genes act in concert to sustain epidermal integrity and mucus production in a terrestrial setting.

To further probe stress‐responsive regulation, we examined transcriptional changes of selected genes in skin and muscle under salt stress (Figure 5C). *MEX3*, *GPCR*, and *SULT1B1* showed significant expression differences in both tissues, indicating shared roles in systemic stress responses, such as signal transduction, neuromodulation, and detoxification. In contrast, *ASGR2*, *COL6A6*, and *LINE‐1* were significantly altered only in the skin. This pattern suggests that these genes participate in skin‐specific adaptations, including extracellular matrix organization, receptor‐mediated uptake, and genomic or epigenomic plasticity in surface tissues that directly face osmotic and mechanical stress. No significant changes were detected for these genes in muscle, highlighting tissue‐specific regulatory control. Conversely, *CPCT1* and *CPCT2* did not change significantly in skin but were differentially expressed in muscle, implying a distinct role in muscle physiology under salt stress, possibly linked to energy use or contractile function.

These histological, spatial expression, and transcriptional data together indicate clear tissue specialization in *L. alte*. The thick, melanized, gland‐rich epidermis, together with the differential expression of vascular, metabolic, and matrix‐related genes in skin, reflects distinct tissue‐level organization in *L. alte*. Together, the histological, FISH, and qPCR results provide tissue‐level support for candidate genes and pathways identified by the multi‐omics analyses. The thick cuticle, abundant mucous glands, and pronounced epidermal pigmentation observed in *L. alte* indicate enhanced protective features of the skin. In addition, tissue‐biased expression patterns of selected genes suggest that the skin is closely associated with stress responses and mucus‐related traits.

### 2.7 VEGF Gene Diversity and Candidate Association With Mucus‐Related Pathways in *L. alte*

**FIGURE 6:**
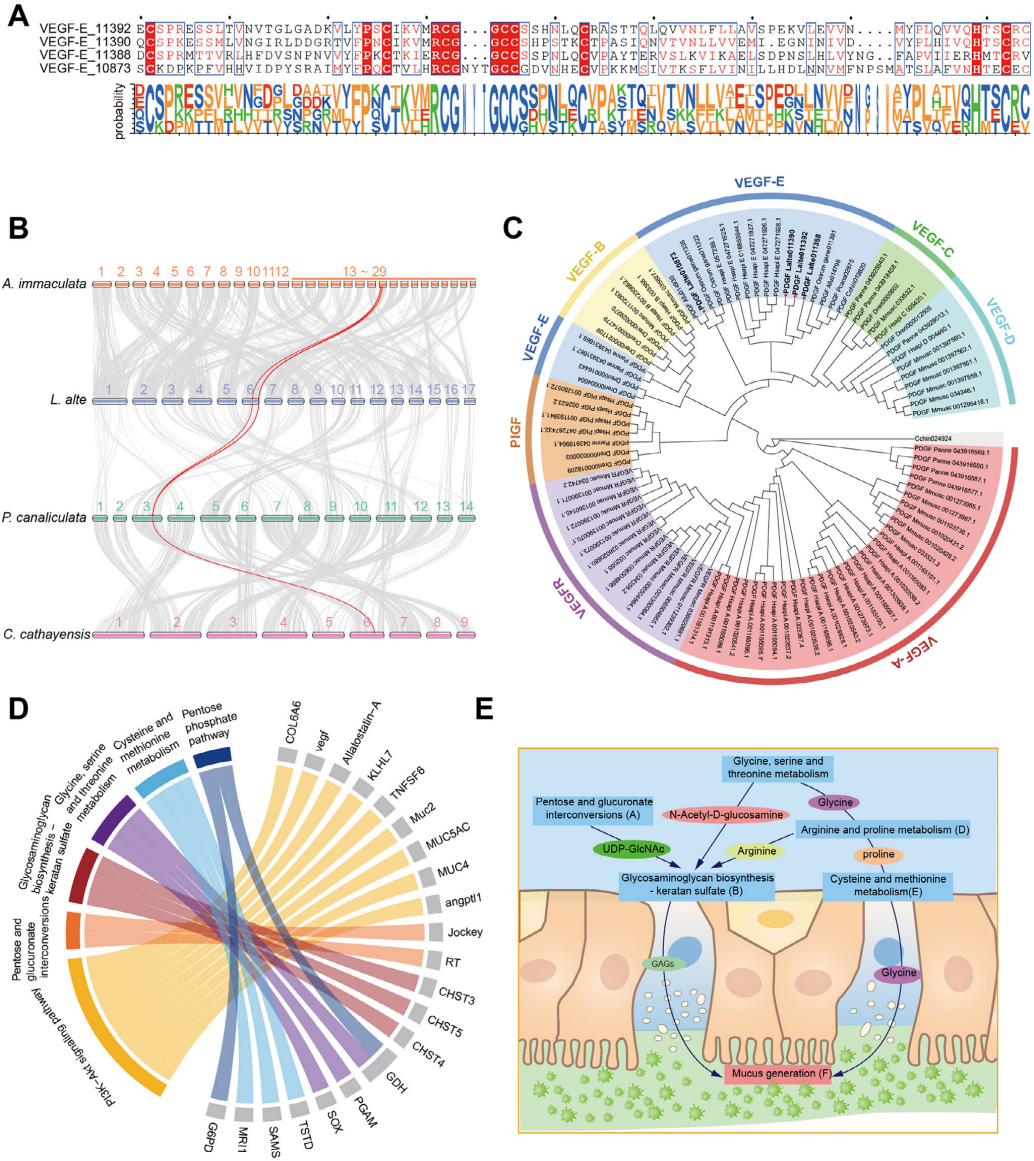
The *VEGF* gene diversity and regulation of mucus production in *Laevicaulis alte*. (A) The sequence alignment and consensus sequence logo for identified VEGF variants in *L. alte*. (B) The genome synteny among *A. immaculata*, *L. alte*, *P. canaliculata*, and *C. cathayensis*. The ends of the red curve are the VEGF for each species. (C) Phylogenetic analysis of VEGFs from *L. alte*, *Onchidium reevesii*, *Pomacea_canaliculata*, *Cipangopaludina chinensis*, *Achatina fulica*, *Danio rerio*, *Protopterus annectens*, *Mus musculus*, and *Homo sapiens*. (D) The chord diagram illustrating the relationship between specific pathways and associated genes. (E) The schematic diagram for possible regulation pathway of mucus generation in *L. alte*.

Among the candidate genes identified above, VEGF showed prominent expression in skin tissue and was associated with pathways related to vascular development and tissue remodeling. Based on these observations, we conducted a focused analysis of the VEGF gene family in *L. alte*. We identified four VEGF genes in *L. alte*, including *VEGF‐E_11392*, *VEGF‐E_11390*, *VEGF‐E_11388*, and *VEGF‐E_10873*. Sequence alignment and sequence logo analysis showed strong evolutionary conservation of key residues in VEGF proteins. Conserved regions mark functional domains essential for VEGF activity, whereas isoform‐specific differences suggest potential functional divergence among paralogs. Highly conserved residues, including cysteine (C), proline (P), and arginine (R), support structural stability and receptor binding (Figure 6A). In particular, the conserved cysteine residues form the classic cysteine‐knot motif, which is critical for dimerization and tertiary structure stabilization in VEGF proteins.

Predicted 3D structures showed that VEGF‐E_11388, VEGF‐E_11390, and VEGF‐E_11392 share very similar folds, while VEGF‐E_10873 displayed a more distinct structure (Figure S9). This structural difference suggests potential functional divergence of VEGF‐E_10873 relative to the other three VEGF‐E paralogs. Synteny analysis indicated that *L. alte* VEGF genes show strong collinearity with those of other gastropods (Figure 6B). This pattern supports conservation of the VEGF gene family across gastropods, with lineage‐specific modifications that may fine‐tune vascular supply to the epidermis and mucus glands in terrestrial species.

Phylogenetic analysis grouped VEGF genes into the canonical subfamilies *VEGF‐A*, *VEGF‐B*, *VEGF‐C*, *VEGF‐D*, *VEGF‐E*, and *PlGF* (placental growth factor) (Figure 6C). *VEGF‐C* and *VEGF‐D* clustered closely, in line with their known roles in lymphangiogenesis. *VEGF* and *PDGF* (platelet‐derived growth factor) genes shared a common ancestor, highlighting their structural and functional similarity. Divergence into these subfamilies supports distinct functions, such as angiogenesis (*VEGF‐A*), lymphangiogenesis (*VEGF‐C/D*), and stress or tissue‐repair responses (*PlGF*). All four *L. alte* VEGF genes fall within the *VEGF‐E* subfamily. This result indicates that the *L. alte* VEGF family is dominated by VEGF‐E‐like members.

A chord diagram summarized the interactions between metabolic pathways and key genes and emphasized the association of VEGF with tissue integrity‐ and angiogenesis‐related pathways (Figure 6D). Pathways such as glycosaminoglycan (GAG) biosynthesis and PI3K‐Akt signaling were strongly linked to structural and metabolic genes, including *COL6A6*, *VEGF*, *MUC4*, *CHST3*, *CHST4*, and *CHST5*. These pathway–gene associations indicate that VEGF is associated with a network enriched for ECM‐, GAG‐, and mucus‐related genes, suggesting a potential connection between vascular signaling and mucus‐associated tissue characteristics.

We then integrated metabolic and signaling data to outline a putative regulatory pathway for mucus generation in *L. alte* (Figure 6E). Mucus production starts with core metabolic routes such as pentose and glucuronate interconversions, which produce UDP‐GlcNAc, a key precursor for GAG biosynthesis. In parallel, glycine, serine, and threonine metabolism supplies glycine and other amino acids required for GAGs and mucins. Arginine and proline metabolism provides proline for structural proteins and arginine for cell growth and maintenance of the mucosal barrier. Cysteine and methionine metabolism contributes sulfur‐containing compounds that stabilize proteins and enzymes essential for mucus synthesis. These precursors feed into GAG biosynthesis pathways that generate keratan sulfate and other GAGs, which are then modified by sulfotransferases.

GAGs combine with mucins, which are heavily glycosylated glycoproteins produced by epithelial cells, to form the hydrated gel structure of functional mucus. Signaling pathways such as PI3K‐Akt support cell survival, regulate secretory activity, and promote mucin and GAG synthesis. Once secreted, the mucus layer acts as a protective barrier that traps pathogens, maintains hydration, and lubricates the slug's surface. Together, these results identify a candidate VEGF‐associated network involving GAG biosynthesis, ECM‐related genes, PI3K–Akt signaling, and mucus‐associated components in *L. alte*. This framework provides a basis for integrating the tissue‐level and multi‐omics results described above.

## 3 Discussion

The integration of high‐throughput multi‐omics approaches has greatly improved our understanding of the molecular mechanisms that underlie adaptation in non‐model organisms [24, 25]. In this study, we focused on *L. alte*, a terrestrial slug with distinct genomic, physiological, and tissue‐level features. We examined how genome organization, gene family evolution, stress‐responsive transcription, metabolite profiles, and tissue differentiation may contribute to terrestrial life. By combining a high‐quality, chromosome‐scale genome with gene family analyses, transcriptomic responses to salinity stress, metabolomic profiles, and histological comparisons, we provide an integrated view of its adaptive strategies. We also placed these results in a comparative framework with other molluscs and model species, to better understand the general evolutionary processes that shape adaptive traits.

The genome survey of *L. alte* revealed very low heterozygosity, indicating a highly homogeneous genome. Compared with several previously published gastropod genomes, such as *A. fulica* [14] and *Lottia gigantea* [26], the chromosome‐scale assembly generated here provides an improved genomic resource for studying terrestrial slug biology. The low heterozygosity of *L. alte* contrasts with marine broadcast spawners and many vertebrates, which typically show higher heterozygosity due to large effective population sizes and extensive gene flow [27]. Instead, *L. alte* resembles species that have experienced strong bottlenecks or self‐fertilization, where reduced genetic diversity is often offset by phenotypic plasticity and specialized gene regulatory systems [28]. In *L. alte*, adaptive innovation may rely less on standing genetic variation and more on changes in gene regulation, structural rearrangements, or bursts of transposable element (TE) activity. These mechanisms can generate functional novelty even in a genetically uniform background. The low heterozygosity may also reflect past demographic events, such as bottlenecks or founder effects, that shaped its genomic landscape [29]. This strategy differs from that of highly diverse vertebrate lineages, which rely on broad genetic repertoires to face fluctuating environments [30, 31].

A notable feature of *L. alte* genome is its putative holocentric chromosomal organization. Hi‐C contact maps and entropy analyses suggest that *L. alte* chromosomes may lack a single, localized centromere and instead show distributed centromeric activity along their length. Such holocentric chromosomes are rare in molluscs and vertebrates but have been described in groups such as Lepidoptera and nematodes [32, 33]. Holocentric chromosomes can better tolerate chromosomal breaks and rearrangements because fragments still carry centromeric activity and can segregate correctly during cell division [32, 34]. This architecture favors rapid karyotype evolution and may promote adaptive genome reshuffling. Holocentricity has been linked to fast karyotypic change and may contribute to speciation in several lineages [35, 36]. In *L. alte*, a holocentric arrangement could provide an evolutionary advantage by allowing flexible structural change while preserving chromosome segregation fidelity, although direct cytogenetic confirmation is still needed.

TEs play a central role in genome evolution, especially in species with low heterozygosity [37]. In *L. alte*, we detected signatures of both recent and ancient TE activity. The TE insertion time distribution showed a recent burst around 0.1 million years ago, superimposed on a background of older, more degenerated insertions. TEs are known to drive genome expansion, structural rearrangements, and the origin of new regulatory and coding sequences [38]. In *L. alte*, the recent TE activity may have increased genomic variability and provided raw material for regulatory or structural changes and local reorganization of regions involved in stress responses. This process could compensate for low standing genetic variation and help the species cope with environmental fluctuations. The coexistence of recent and ancient TE layers reflects a dynamic balance between stability and variability, a pattern also reported in other molluscs such as *Crassostrea ariakensis* [39], *Saccostrea glomerata* [40], and *Crassostrea gigas* [41], where TEs have been linked to environmental adaptation. In *L. alte*, TE‐driven plasticity may similarly underpin rapid evolutionary responses.

We identified marked expansions of several gene families in *L. alte*, particularly those linked to ECM–receptor interaction, PI3K‐Akt signaling, and neuroactive ligand‐receptor interaction. These expansions likely represent lineage‐specific innovations that support key adaptive traits such as enhanced tissue repair, improved structural integrity, and refined stress responses [42, 43]. The expansion of ECM–receptor interaction pathways is notable in the context of tissue regeneration and barrier maintenance [44]. ECM components and their receptors are crucial for cell adhesion, migration, and wound healing, which are vital in organisms that frequently experience mechanical abrasion and osmotic stress [45, 46]. In *L. alte*, these expansions may underlie the formation of a thick cuticular membrane and dense mucus‐producing epidermis, both essential for desiccation resistance and pathogen defense. Likewise, the expansion of PI3K‐Akt signaling points to reinforced mechanisms for cell survival, growth, and metabolic control [47]. In vertebrates, this pathway supports stress resilience and longevity, and similar advantages may apply in *L. alte*. Comparative analyses with other molluscs, such as *P. canaliculata*, show conserved syntenic blocks, yet *L. alte* displays more extensive gene family expansions [48]. This suggests that shared ancestral genomic frameworks were subsequently modified by lineage‐specific duplications and rearrangements to meet the ecological needs of *L. alte*.

Metabolomic profiling showed substantial metabolic reprogramming in *L. alte* mucus under environmental challenges. Among 379 detected metabolites, 120 changed significantly, with most being upregulated. Enriched pathways included amino acid biosynthesis, arginine and proline metabolism, and cofactor biosynthesis. Notably, pathways related to cutin, suberin, and wax biosynthesis were strongly enriched. These metabolic routes produce barrier‐forming compounds that reduce water loss, limit physical damage, and restrict microbial invasion. Similar strategies are seen in terrestrial plants that increase wax and suberized compounds to resist desiccation and pathogens, and in insects that develop protective cuticles to prevent water loss [49, 50]. *L. alte* appears to have converged on comparable metabolic solutions for barrier reinforcement. KEGG enrichment further showed upregulation of sphingolipid metabolism and alkaloid biosynthesis. Sphingolipids are critical for membrane integrity and can stabilize cells under osmotic or thermal stress [51, 52], while alkaloids often function as chemical defenses against predators and pathogens [53]. In *L. alte*, these metabolic changes complement structural traits such as the thick cuticular membrane and increased mucus gland density, together forming a multi‐layered defense system.

Among the many gene families examined, the VEGF family is of particular interest because VEGF proteins are widely involved in tissue repair and vascular‐related processes in tissue repair and vascular development [54, 55]. We identified four VEGF genes in *L. alte* that share conserved motifs, including the cysteine‐knot, but show isoform‐specific structural variation [56]. Conserved residues such as cysteine, proline, and arginine help maintain structural integrity, dimerization, and receptor‐binding properties that are essential for biological function. Comparative analyses show that VEGF genes are conserved across metazoans, but the extent of diversification and specialization differs among lineages. In vertebrates, distinct VEGF isoforms mediate angiogenesis, lymphangiogenesis, and neuroprotection [55, 57]. *VEGF‐A* primarily promotes blood vessel growth, while *VEGF‐C* and *VEGF‐D* are key regulators of lymphatic vessel formation [58, 59].

The phylogenetic placement of *L. alte* VEGF genes alongside vertebrate VEGF subfamilies underscores the deep evolutionary conservation of this protein family despite large phylogenetic distances. This pattern suggests that similar selective pressures, such as the need for efficient tissue repair and vascular support, have favored both conservation and diversification of VEGF across animal phyla. Integration of VEGF signaling with ECM production and metabolic regulation further highlights its central role in maintaining tissue integrity. Chord diagrams linking *VEGF*, *COL6A6*, *MUC4*, and metabolic pathways suggest a potential connection between tissue remodeling, extracellular matrix organization, and mucus‐associated surface protection. These associations suggest that VEGF‐related genes may participate in broader tissue‐maintenance and stress‐response networks in *L. alte*, but functional validation is needed to confirm their specific roles.

Histological comparisons between *L. alte* and *O. reevesii* reveal clear skin adaptations that match their ecological niches. As a terrestrial slug, *L. alte* has evolved features that promote desiccation resistance and mechanical protection, whereas the semi‐terrestrial *O. reevesii* is adapted to moist, intertidal conditions [60]. Dermal gland composition differs between the two species, *O. reevesii* has both granular and mucus glands; granular glands secrete materials for moisture retention and locomotion, and numerous small mucus glands reduce friction in aquatic or semi‐aquatic environments [61]. In contrast, granular glands were not evident in the examined *L. alte* sections and relies on densely packed mucus glands as its primary mechanism for water retention and surface protection. Both species possess dermal fibers and immune cells that contribute to skin integrity and pathogen defense [62]. *L. alte* also has abundant melanocytes, which likely provide UV protection and may aid in thermoregulation [63].

Ventral fold morphology reflects their different lifestyles: *L. alte* has shorter, denser folds that enhance traction and moisture retention on land, whereas *O. reevesii* has longer, sparser folds that facilitate water exchange and reduce drag. Comparisons with other gastropods support these patterns, the terrestrial *A. fulica* also has a thick cuticular membrane that protects against desiccation [64], while aquatic *Lymnaea stagnalis* has thinner cuticle, fewer mucus glands [65], and little pigmentation because UV exposure is lower underwater [66]. Together, these structural differences illustrate how closely skin architecture and mucus systems track habitat demands.

## 4 Conclusion

This study presents the first T2T genome assembly of *L. alte*. Low heterozygosity and a possible holocentric chromosome structure set the genomic context for gene family expansions and dynamic TE activity, which together create an evolvable yet stable genome. Gene family expansions, especially in ECM‐related and stress signaling pathways, suggest enhanced tissue resilience and cellular survival. Metabolomic reprogramming in pathways related to barrier formation, lipid metabolism, and chemical defense reflects active biochemical remodeling in response to environmental stress. Transcriptomic responses highlight key genes involved in immune modulation, ion transport, and energy balance, particularly under salinity stress. Histological traits such as a thick cuticular membrane, dense mucus glands, and abundant melanocytes provide visible evidence of these molecular adaptations.

Taken together, these results show that *L. alte* relies on a tightly integrated system that links a gap‐free genome, flexible gene networks, metabolic plasticity, and specialized tissues to support terrestrial adaptation and high mucus output. When viewed in a broader evolutionary context, the strategies seen in *L. alte* echo themes found in other taxa, underscoring the general importance of genomic plasticity, metabolic flexibility, and efficient tissue repair in the evolution of adaptive traits.

## 5 Methods

### 5.1 Sample Collection for Genome Sequencing

Adult *L. alte* were collected from Tangkou Town, Yangxi District, Yangjiang City, Guangdong Province, China (21.8789°N, 111.6165°E). The specimen used for this study was a hermaphroditic adult, measuring 7.20 cm in length and 1.61 cm in width. The ventral foot tissue of *L. alte* was harvested, immediately frozen in liquid nitrogen, and stored at −80°C.

### 5.2 DNA Library Preparation and Sequencing

Genomic DNA (gDNA) was extracted by a modified CTAB method [67]. Briefly, to accommodate the mucopolysaccharide‐rich foot tissue of *L. alte*, the protocol was modified by adding a potassium acetate precipitation step (1/3 volume of 5 m KAc, pH 5.2, ice 30 min) after chloroform extraction to remove mucus and polysaccharides, followed by isopropanol precipitation, DNase‐free RNase A treatment (100 µg mL^−1^, 37°C, 30 min), and two additional rounds of chloroform:isoamyl alcohol (24:1) extraction to improve DNA purity. The quality and quantity of the extracted gDNA were assessed using a spectrophotometer (Thermo, NanoDrop 2000), a fluorometer (Thermo, Qubit 3.0), and by electrophoresis on a 0.8% agarose gel. Pair‐end libraries (insert size = 350 bp) were constructed for sequencing to obtain Illumina short reads. The DNA library for HiFi sequencing were constructed using high quality gDNA and SMRTbell express template prep 2.0 (Pacbio). The Hi‐C library was preprocessed using the NEBNext Ultra II DNA Library Prep Kit for Illumina (NEB, E7645S). For the DNA library utilized in Nanopore sequencing, processing was conducted with the Ligation Sequencing Kit (ONT, SQK‐LSK109). The sequencing work was completed by Frasergen Biotechnology Co., Ltd (Wuhan, China).

### 5.3 Genome Survey and Assembly

Initial filtering of sequencing data was performed using the fastp (v0.23.2), which removed adapter, contaminants, and low‐quality sequences. The quality‐controlled Illumina short reads will be used for genome survey. The 21‐mer frequency distribution followed a Poisson distribution, from which the peak depth was identified, representing the average depth and variation of the genome. The genome size and K‐mer depth distribution were calculated using Jellyfish software (v2.2.4) [68].

HiFi reads were generated using CCS software (v6.4.0) with the parameter “‐minPasses 3.” The Hifiasm (v0.20.0) [69] was used to combine HiFi reads and ultra‐long reads generated by Nanopore sequencing to generate preliminary genome assembly. Error correction of the primary assembly was performed using short Illumina reads through the Pilon tool (v1.23) [70].

Hi‐C data were further screened and quality controlled using HICUP (v0.9.2). Paired reads with unique alignments at both ends were retained, while those more than 500 bp from restriction sites or containing errors such as circularization and fragment size discrepancies were removed. Valid reads were input into the Juicer (v1.5.6) to construct an unmounted genome interaction matrix, which was then processed with the 3D‐DNA software to scaffold the genome. To resolve assembly gaps, 10 kb sequences flanking the gaps were extracted and aligned to contigs from ultra‐long Nanopore data. Alignment parameters included an E‐value of 1e‐10, similarity threshold of 99.9%, and continuous alignment length over 9 kb. Only unique alignments matching gap flanking sequences in position and orientation were used for gap filling. Additionally, 10 kb sequences from the ends of chromosomes were aligned with contigs to extend and complete the chromosomes based on the alignment results. The completeness of the genome assembly was evaluated using BUSCO (v5.8.2) with mollusca_odb10 and metazoa_odb10.

### 5.4 Repeat Sequence and Non‐Coding Sequence Annotation

Tandem repeats were identified using the TRF tool (v4.09) [71], while microsatellites were detected with the misa.pl script (Lowe and Eddy, 1997). Long terminal repeats (LTRs) were first identified using LTR_finder and LTR_harvest [72, 73], and the final LTR annotations were refined by integrating the results from both tools through LTR_retriever (v2.7) [74]. Following LTR refinement with LTR_retriever, insertion times of intact LTR retrotransposons were estimated based on the sequence divergence between paired 5′ and 3′ LTRs. Because the two LTRs are identical at the time of insertion and subsequently accumulate mutations independently, insertion time was calculated as T = K / 2r, where K represents the sequence divergence between the 5′ and 3′ LTRs and r is the neutral substitution rate. A neutral substitution rate of 1 × 10^−^^9^ substitutions per site per year was used to convert divergence values into absolute insertion times. The resulting ages were converted to million years ago (MYA). To visualize the temporal dynamics of LTR amplification, kernel density estimation was performed on the insertion time data using the density function in R, and the distribution was plotted with the ggplot2 package. Short Interspersed Nuclear Elements (SINEs), Long Interspersed Nuclear Elements (LINEs), and DNA transposons were annotated using RepeatMasker (v4.0.7) [75].

Non‐coding RNA (ncRNA) includes RNA molecules that do not code for proteins, such as ribosomal RNA (rRNA), transfer RNA (tRNA), small nuclear RNA (snRNA), and small nucleolar RNA (snoRNA), all of which perform crucial biological functions. Specifically, tRNA and rRNA are essential for protein synthesis, while snRNA and snoRNA are involved in RNA precursor processing and splicing. In this study, The identification of rRNA was performed using RNAMMER (v1.2) [76], while the prediction of tRNA was conducted with tRNAscan‐SE (v1.3.1) [77]. The detection of small nuclear RNA (snRNA) and small nucleolar RNA (snoRNA) was done using cmscan with the Rfam14.0 database [78].

### 5.5 Protein‐Coding Gene Annotation

RNA sequencing (RNA‐seq) was performed on multiple tissues of *L. alte*, including gills, kidneys, skin, heart, gonads, intestines, and muscle. The mixed RNA‐seq data from these tissues were subsequently used for gene structure prediction. Initially, MAKER2 (v2.31.10) [79] was employed to conduct the first‐round gene prediction, incorporating both protein sequences from closely related species and transcriptome sequences derived from the *L. alte* itself. The resulting gene structure models from MAKER2 were used to train a species‐specific model using AUGUSTUS software [80], which was then applied for *de novo* gene structure prediction. The gene structures generated from these two methods were subsequently integrated using the Evidence Modeler (EVM) tool [81] to produce the final gene models.

### 5.6 Gene Functional Annotation

Gene function annotation was performed by aligning the predicted protein sequences against several major protein databases, including the NCBI Non‐Redundant (NR) database, TrEMBL, KOG, and Swiss‐Prot, using BLASTP (v2.6.0) with an *E*‐value threshold of 1e‐5. Additional functional annotation was achieved by mapping the proteins to the KEGG database. Protein domains were identified through PfamScan (v1.6.0) and the Pfam database, while GO IDs were assigned to each gene using Blast2GO for further functional classification.

### 5.7 Comparative Genomic Analysis

Gene family clustering was conducted using the OrthoFinder program. Homologous gene pairs were identified by comparing all protein sequences with the Diamond BLASTP program, applying a *E*‐value ≤ 1e^−5^ and minimum coverage of 40%. The resulting homologous gene pairs were then processed through the MCL program for family clustering, and gene family data was extracted and organized for further analysis. For the phylogenetic analysis, single‐copy orthologous proteins were aligned using the MUSCLE program. The alignment results were standardized and converted into MEGA format. Using MEGA software, an evolutionary tree was constructed based on the neighbor‐joining (NJ) method with 1000 bootstrap replicates. Divergence times were estimated using the approximate likelihood method implemented in mcmctree [82]. Branch length gradient and Hessian matrices were first estimated under the HKY85 + Γ substitution model with five discrete gamma categories and a shape parameter alpha of 0.5. The Bayesian divergence time estimation was performed using usedata = 2. We employed an independent rates model (clock = 2). The MCMC chain was run for a total of 14 million generations (burnin = 4 000 000, sampfreq = 100, nsample = 100 000). The species divergence times were calibrated using fossil data from the TimeTree database. Specifically, calibration priors were assigned to the following nodes: 1) *Corbicula fluminea* and *Lottia gigantea* (95% CI: 515.0–541.7 MYA); 2) *Marisa cornuarietis* and *Chrysomallon squamiferum* (95% CI: 379.7–500.0 MYA); 3) *Aplysia californica* and *C. unifasciata* (95% CI: 58.3–278.9 MYA); 4) *Peronia verruculata* and *C. unifasciata* (95% CI: 19.7–255.0 MYA). The iTOL online tool was used to visually enhance and annotate the evolutionary tree.

The gene family clustering data was further analyzed to identify common gene families across all species and unique gene families for each species. For gene family contraction and expansion analysis, the phylogenetic data and gene family clustering results were input into the CAFE (v5.1.0) to identify significant contractions or expansions at the species level and key divergence nodes, with a *p*‐value < 0.05 indicating statistical significance.

### 5.8 Metabolomics Study

Samples of mucus from *L. alte* and *P. verruculata* were taken for metabolomic profiling. For each 200 µL of sample, 800 µL of extraction solution (methanol: acetonitrile, 1:1 v/v) containing 0.02 mg mL^−1^ of the internal standard (L‐2‐chlorophenylalanine) was added, and then ultrasound extracted at 5°C and 40 KHz. The supernatant was dried under nitrogen gas, and 100 µL of reconstitution solution (acetonitrile:water, 1:1 v/v) was added. The mixture was vortexed and subjected to another round of low‐temperature ultrasound extraction. The LC‐MS was performed on an UHPLC‐Q Exactive HF‐X system (Thermo Fisher Scientific, USA).

The raw data were imported into the Progenesis QI software (Waters Corporation, CT, USA) for baseline filtering, peak identification, integration, retention time correction, and peak alignment. Subsequently, the variables with non‐zero values in more than 80% of any sample group were retained. Missing values were filled using 1/2 of the minimum value from the original matrix. Data normalization was conducted using the total peak area normalization method, and variables with a relative standard deviation (RSD) of QC samples ≥30% were excluded. The MS and MS/MS mass spectrometry data were matched against metabolic databases, including commercial databases like HMDB (http://www.hmdb.ca/), Metlin (https://metlin.scripps.edu/), as well as public and self‐constructed databases for metabolite identification.

The t‐test, combined with the multivariate analysis method OPLS‐DA, was used to identify differential metabolites between groups, with the criteria set to VIP > 1 and *p*‐value < 0.05. Integration of information from HMDB, KEGG Compound Database and LIPID MAPS to annotate these metabolites and perform pathway analysis.

### 5.9 Comparative Transcriptomic Study

Experimental individuals were placed in Petri dishes lined with filter paper moistened with 1% (w/v) NaCl solution and subjected to short‐term hyperosmotic stress for 2 h to simulate osmotic challenge under a humid substrate environment. Control individuals were maintained under identical conditions but without NaCl treatment. At the end of the exposure period, tissues were immediately collected for total RNA extraction. Total RNA was extracted from the samples using the Trizol method. Qualified RNA was purified using poly‐T oligo‐attached magnetic beads. Sequencing libraries were constructed using the VAHTS Universal V6 RNA‐seq Library Prep Kit for MGI (Vazyme, NRM604‐01). Sequencing was carried out on the MGISEQ‐2000 platform.

The Bowtie2 software was utilized to align the sequencing reads to the reference genes [83]. Read counts for each sample were processed using the DESeq2 (v1.48.1) [84]. Gene expression levels were quantified using FPKM, with differentially expressed genes (DEGs) defined by a q‐value < 0.05 and |log2(fold change)| > 1. Functional enrichment analysis of the DEGs was performed using the clusterProfiler (v4.12.6) [85].

### 5.10 Quantitative Analysis of Gene Expression via qPCR

Reverse transcription employed the PrimeScript RT Reagent Kit with gDNA eraser (Takara, RR047Q) to synthesize cDNA first strands. Amplification of genes was performed using gene‐specific forward and reverse primers (Table S10). The qPCR reactions were conducted in 96‐well plates with 20 µL reactions comprising 10 µL SYBR Premix EX Taq II (Takara, DRR041A), 0.4 µL each primer, 2 µL cDNA template, and 7.2 µL sterile ddH2O. Thermocycling conditions were: 95°C for 5 min, 40 cycles of 95°C for 10 s and 72°C for 15 s. Relative expression levels were calculated using the 2^−ΔΔCt^ method [86].

### 5.11 Histological Observation

Skin samples from *L. alte* were carefully collected following a brief anesthetization by exposure to ice for 3 min. The skins were then excised and immediately fixed in a 4% paraformaldehyde solution. Afterward, the specimens were thoroughly cleaned before being embedded in paraffin wax. Thin sections, measuring 6 µm in thickness, were precisely prepared using a microtome and carefully mounted onto glass slides for microscopic examination. The slides were counterstained with hematoxylin‐eosin. The stained sections were then examined under a microscope (Olympus, BX63).

### 5.12 Fluorescence In Situ Hybridization

Skin and foot samples of *L. alte* were taken and stored in 4% paraformaldehyde for fixation. Serial sections were made at a thickness of 5 µm. Sections were deparaffinized, rehydrated, and incubated with 4 µg mL^−1^ proteinase K (Roche, Mannheim, Germany) at 37°C for 15 min. Subsequently, the samples were hybridized overnight at 60°C with a specific RNA probe (Table S11). Post‐hybridization washes were performed by successive 30 min incubations in 50% formamide/2X SSC, 2X SSC, and 0.2X SSC. Sections were subsequently incubated for 30 min at room temperature with Anti‐DIG‐POD (1:200 dilution in DIG2 buffer) in a humidified chamber, washed in DIG1 buffer, and hybridization signals amplified using the TSA Plus fluorescein detection kit (Akoya Biosciences, NEL701A001KT). Nuclei were counterstained with DAPI prior to fluorescence imaging on a fluorescence microscope (Olympus, BX63).

## Author Contributions

G.W., L.C., D.Z., and S.H. conceived the project. S.C., Z.W., R.L., F.W., L.W., and N.S. performed experiments. X.S., Z.B., S.Z., D.W., and Y.S. analyzed data. G.W., A.P., and K.T. drafted the manuscript. B.T., Y.F., and Q.J. supervised the research. All authors read and approved the final manuscript.

## Conflicts of Interest

The authors declare no competing interests.

## Supporting information




**Supporting File 1**: advs76129‐sup‐0001‐SuppMat.pdf.

**Supporting File 2**: advs76129‐sup‐0002‐TableS1‐S11.xlsx.

## Data Availability

The raw sequencing data generated during the current study are available in the China National Center for Bioinformation repository [PRJCA039302]. The genome annotation file generated in this study have been deposited in Figshare and are publicly accessible at: https://doi.org/10.6084/m9.figshare.32056083.

## References

[1] M. Liegertová and J. Malý , “Gastropod Mucus: Interdisciplinary Perspectives on Biological Activities, Applications, and Strategic Priorities,” ACS Biomaterials Science & Engineering 9, no. 10 (2023): 5567–5579, 10.1021/acsbiomaterials.3c01096. 37751898 PMC10566510

[2] T. P. T. Ng , S. H. Saltin , M. S. Davies , K. Johannesson , R. Stafford , and G. A. Williams , “Snails and Their Trails: The Multiple Functions of Trail‐Following in Gastropods,” Biological Reviews 88, no. 3 (2013): 683–700, 10.1111/brv.12023. 23374161

[3] C. Avila , L. Núñez‐Pons , and J. Moles , “From the Tropics to the Poles: Chemical Defense Strategies in Sea Slugs (Mollusca: Heterobranchia),” in Chemical Ecology: The Ecological Impacts of Marine Natural Products, (CRC Press, 2018), 71–163, 10.1201/9780429453465.

[4] P. A. Rühs , J. Bergfreund , P. Bertsch , et al., “Complex Fluids in Animal Survival Strategies,” Soft Matter 17, no. 11 (2021): 3022–3036. 33729256 10.1039/d1sm00142f

[5] P. Fischer , “Sand and Mucus: A Toolbox for Animal Survival,” Physics Today 75, no. 12 (2022): 30–37, 10.1063/PT.3.5137.

[6] J. Newar , S. Verma , and A. Ghatak , “Effect of Metals on Underwater Adhesion of Gastropod Adhesive Mucus,” ACS Omega 6, no. 6 (2021): 15580–15589, 10.1021/acsomega.0c06132. 34179602 PMC8223214

[7] M. Rashad , S. Sampò , A. Cataldi , and S. Zara , “Biological Activities of Gastropods Secretions: Snail and Slug Slime,” Natural Products and Bioprospecting 13, no. 1 (2023): 42, 10.1007/s13659-023-00404-0. 37870705 PMC10593653

[8] M. McDermott , A. R. Cerullo , and J. Parziale , “Advancing Discovery of Snail Mucins Function and Application,” Frontiers in Bioengineering and Biotechnology 9 (2021): 734023, 10.3389/fbioe.2021.734023. 34708024 PMC8542881

[9] A. R. Cerullo , M. B. McDermott , and L. E. Pepi , “Comparative Mucomic Analysis of Three Functionally Distinct Cornu Aspersum Secretions,” Nature Communications 14, no. 1 (2023): 5361, 10.1038/s41467-023-41094-z. PMC10475054 37660066

[10] J. Melrose , “High Performance Marine and Terrestrial Bioadhesives and the Biomedical Applications They Have Inspired,” Molecules (Basel, Switzerland) 27, no. 24 (2022): 8982, 10.3390/molecules27248982. 36558114 PMC9783952

[11] Y. Lu , X. Xu , and J. Li , “Recent Advances in Adhesive Materials Used in the Biomedical Field: Adhesive Properties, Mechanism, and Applications,” Journal of Materials Chemistry B 11, no. 15 (2023): 3338–3355, 10.1039/D3TB00251A. 36987937

[12] W. F. Ponder , D. R. Lindberg , and J. M. Ponder , Biology and Evolution of the Mollusca, Vol. 1 (CRC Press, 2019), 924.

[13] A. F. Torres , O. S. Wangensteen , W. Renema , C. P. Meyer , I. K. C. Fontanilla , and J. A. Todd , “Global Species Hotspots and COI Barcoding Cold Spots of Marine Gastropoda,” Biodiversity and Conservation 33, no. 10 (2024): 2925–2947, 10.1007/s10531-024-02896-9.

[14] Y. Guo , Y. Zhang , Q. Liu , et al., “A Chromosomal‐Level Genome Assembly for the Giant African Snail Achatina fulica,” GigaScience 8, no. 10 (2019): giz124. 31634388 10.1093/gigascience/giz124 PMC6802634

[15] C. Liu , Y. Ren , and Z. Li , “Giant African Snail Genomes Provide Insights into Molluscan Whole‐Genome Duplication and Aquatic–terrestrial Transition,” Molecular Ecology Resources 21, no. 2 (2021): 478–494, 10.1111/1755-0998.13261. 33000522

[16] Z. Chen , Ö. Doğan , N. Guiglielmoni , et al., “Pulmonate Slug Evolution Is Reflected in the De Novo Genome of Arion vulgaris Moquin‐Tandon, 1855,” Scientific Reports 12, no. 1 (2022): 14226. 35987814 10.1038/s41598-022-18099-7 PMC9392753

[17] S. Sun , X. Han , Z. Han , et al., “Chromosomal‐Scale Genome Assembly and Annotation of the Land Slug (Meghimatium bilineatum),” Scientific Data 11, no. 1 (2024): 35. 38182611 10.1038/s41597-023-02893-7 PMC10770140

[18] R. F. Ali and D. G. Robinson , “Recording the Terrestrial Slug Species Laevicaulis alte (Férussac, 1822) (Pulmonata: Veronicellidae) in Ornamental Plants Nursery in Giza Governorate, Egypt,” Universal Journal of Agricultural Research 10, no. 2 (2022): 170–174, 10.13189/ujar.2022.100208.

[19] P. B. Budha , F. Naggs , and T. Backeljau , “Annotated Checklist of the Terrestrial Gastropods of Nepal,” ZooKeys 492 (2015): 1–48, 10.3897/zookeys.492.9175. PMC4389214 25878541

[20] D. G. Reid , N. A. Aravind , and N. A. Madhyastha , “A Unique Radiation of Marine Littorinid Snails in the Freshwater Streams of the Western Ghats of India: The Genus Cremnoconchus W.T. Blanford, 1869 (Gastropoda: Littorinidae),” Zoological Journal of the Linnean Society 167, no. 1 (2013): 93–135, 10.1111/j.1096-3642.2012.00875.x.

[21] M. K. Bhavare and S. R. Magare , “Ecology and Population Studies of Land Slug, laevicaulis alte in shahada region,” Journal of Applied and Advanced Research 2, no. 2 (2017): 63–66, 10.21839/jaar.2017.v2i2.58.

[22] N. V. Subba Rao , S. C. Mitra , S. Barua , et al., “Food Preference, Growth Rate and Fecundity of the Garden Slug *Laevicaulis alte* ,” Environment and Ecology (Kalyani) 7, no. 1 (1989): 211–214.

[23] D. W. Foltz , H. Ochman , J. S. Jones , S. M. Evangelisti , and R. K. Selander , “Genetic Population Structure and Breeding Systems in Arionid Slugs (Mollusca: Pulmonata),” Biological Journal of the Linnean Society 17, no. 3 (1982): 225–241, 10.1111/j.1095-8312.1982.tb02018.x.

[24] M. S. Clark , J. I. Hoffman , and L. S. Peck , “Multi‐Omics for Studying and Understanding Polar Life,” Nature Communications 14, no. 1 (2023): 7451, 10.1038/s41467-023-43209-y. PMC10656552 37978186

[25] H. Li and R. Durbin , “Genome Assembly in the Telomere‐to‐Telomere Era,” Nature Reviews Genetics 25, no. 9 (2024): 658–670, 10.1038/s41576-024-00718-w. 38649458

[26] O. Simakov , F. Marletaz , and S.‐J. Cho , “Insights into Bilaterian Evolution from Three Spiralian Genomes,” Nature 493, no. 7433 (2013): 526–531, 10.1038/nature11696. 23254933 PMC4085046

[27] G. H. Pogson , “Studying the Genetic Basis of Speciation in High Gene Flow Marine Invertebrates,” Current Zoology 62, no. 6 (2016): 643–653, 10.1093/cz/zow093. 29491951 PMC5804258

[28] H. S. Callahan , H. Maughan , and U. K. Steiner , “Phenotypic Plasticity, Costs of Phenotypes, and Costs of Plasticity,” Annals of the New York Academy of Sciences 1133 (2008): 44–66, 10.1196/annals.1438.008. 18559815

[29] N. Balkenhol , R. Y. Dudaniec , and K. V. Krutovsky , “Landscape Genomics: Understanding Relationships Between Environmental Heterogeneity and Genomic Characteristics of Populations,” in Population Genomics: Concepts, Approaches and Applications, ed. O. P. Rajora (Springer International Publishing, 2017), 261–322.

[30] A. Bendesky and C. I. Bargmann , “Genetic Contributions to Behavioural Diversity at the Gene–Environment Interface,” Nature Reviews Genetics 12, no. 12 (2011): 809–820, 10.1038/nrg3065. 22064512

[31] N. Niepoth and A. Bendesky , “How Natural Genetic Variation Shapes Behavior,” Annual Review of Genomics and Human Genetics 21 (2020): 437–463, 10.1146/annurev-genom-111219-080427. 32283949

[32] M. Mandrioli and G. C. Manicardi , “Holocentric Chromosomes,” PLOS Genetics 16, no. 7 (2020): 1008918, 10.1371/journal.pgen.1008918. PMC7392213 32730246

[33] P. M. Carlton , R. E. Davis , and S. Ahmed , “Nematode Chromosomes,” Genetics 221, no. 1 (2022): iyac014, 10.1093/genetics/iyac014. 35323874 PMC9071541

[34] F. Zedek and P. Bureš , “Holocentric Chromosomes: from Tolerance to Fragmentation to Colonization of the Land,” Annals of Botany 121, no. 1 (2018): 9–16, 10.1093/aob/mcx118. 29069342 PMC5786251

[35] P. G. Hofstatter , G. Thangavel , and T. Lux , “Repeat‐Based Holocentromeres Influence Genome Architecture and Karyotype Evolution,” Cell 185, no. 17 (2022): 3153–3168.e18, 10.1016/j.cell.2022.06.045. 35926507

[36] Y. Mata‐Sucre , L. M. Parteka , and C. M. Ritz , “Oligo‐Barcode Illuminates Holocentric Karyotype Evolution in Rhynchospora (Cyperaceae),” Frontiers in Plant Science 15 (2024): 1330927, 10.3389/fpls.2024.1330927. 38384757 PMC10879424

[37] Y. Bourgeois and S. Boissinot , “On the Population Dynamics of Junk: a Review on the Population Genomics of Transposable Elements,” Genes 10, no. 6 (2019): 419, 10.3390/genes10060419. 31151307 PMC6627506

[38] J. L. Bennetzen and H. Wang , “The Contributions of Transposable Elements to the Structure, Function, and Evolution of Plant Genomes,” Annual Review of Plant Biology 65 (2014): 505–530, 10.1146/annurev-arplant-050213-035811. 24579996

[39] G. Zhang , X. Fang , and X. Guo , “The Oyster Genome Reveals Stress Adaptation and Complexity of Shell Formation,” Nature 490, no. 7418 (2012): 49–54, 10.1038/nature11413. 22992520

[40] A. Li , H. Dai , and X. Guo , “Genome of the Estuarine Oyster Provides Insights into Climate Impact and Adaptive Plasticity,” Communications Biology 4, no. 1 (2021): 1287, 10.1038/s42003-021-02823-6. 34773106 PMC8590024

[41] D. Powell , S. Subramanian , S. Suwansa‐Ard , et al., “The Genome of the Oyster Saccostrea Offers Insight into the Environmental Resilience of Bivalves,” DNA Research 25, no. 6 (2018): 655–665. 30295708 10.1093/dnares/dsy032 PMC6289776

[42] D. Huo , S. Liu , L. Zhang , et al., “Importance of the ECM‐Receptor Interaction for Adaptive Response to Hypoxia Based on Integrated Transcription and Translation Analysis,” Molecular Ecology 34 (2024): 17352. 10.1111/mec.17352 38624130

[43] W. Qin , L. Cao , and I. Y. Massey , “Role of PI3K/Akt Signaling Pathway in Cardiac Fibrosis,” Molecular and Cellular Biochemistry 476, no. 11 (2021): 4045–4059, 10.1007/s11010-021-04219-w. 34244974

[44] M. Petreaca and M. Martins‐Green , “Chapter 2 – Cell‐ECM Interactions in Repair and Regeneration,” in Principles of Regenerative Medicine (Second Edition), ed. A. Atala , R. Lanza , J. A. Thomson , (Academic Press, 2011), 19–65.

[45] V. Poltavets , M. Kochetkova , S. M. Pitson , and M. S. Samuel , “The Role of the Extracellular Matrix and Its Molecular and Cellular Regulators in Cancer Cell Plasticity,” Frontiers in Oncology 8 (2018): 431, 10.3389/fonc.2018.00431. 30356678 PMC6189298

[46] D. A. C. Walma and K. M. Yamada , “The Extracellular Matrix in Development,” Development 147, no. 10 (2020): dev175596, 10.1242/dev.175596. 32467294 PMC7272360

[47] A. Samakova , A. Gazova , N. Sabova , S. Valaskova , M. Jurikova , and J. Kyselovic , “The PI3k/Akt Pathway Is Associated with Angiogenesis, Oxidative Stress and Survival of Mesenchymal Stem Cells in Pathophysiologic Condition in Ischemia,” Physiological Research 68, no. Suppl 2 (2019): S131–S138, 10.33549/physiolres.934345. 31842576

[48] S. Farhat , M. V. Modica , and N. Puillandre , “Whole Genome Duplication and Gene Evolution in the Hyperdiverse Venomous Gastropods,” Molecular Biology and Evolution 40, no. 8 (2023): msad171, 10.1093/molbev/msad171. 37494290 PMC10401626

[49] S. J. Vishwanath , C. Delude , F. Domergue , and O. Rowland , “Suberin: Biosynthesis, Regulation, and Polymer Assembly of a Protective Extracellular Barrier,” Plant Cell Reports 34, no. 4 (2015): 573–586, 10.1007/s00299-014-1727-z. 25504271

[50] P. M. Wettstein‐Knowles , “Waxes, Cutin, and Suberin,” in Lipid Metabolism in Plants (CRC Press, 1993).

[51] M. Maceyka and S. Spiegel , “Sphingolipid Metabolites in Inflammatory Disease,” Nature 510, no. 7503 (2014): 58–67, 10.1038/nature13475. 24899305 PMC4320971

[52] E. Iessi , M. Marconi , V. Manganelli , et al., “On the Role of Sphingolipids in Cell Survival and Death,” International Review of Cell and Molecular Biology 351 (2020): 149–195. 32247579 10.1016/bs.ircmb.2020.02.004

[53] L. Dias and J. C , “The Bitter Truth: How Insects Cope with Toxic Plant Alkaloids,” Journal of Experimental Botany 76, no. 1 (2025): 5–15, 10.1093/jxb/erae312. 39028613

[54] S. P , D. Skd , and B. R , “VEGF Signaling: Role in Angiogenesis and beyond,” Biochimica et Biophysica Acta Reviews on Cancer 1879, no. 2 (2024): 189079. 38280470 10.1016/j.bbcan.2024.189079 PMC12927493

[55] A. Ahmad and M. I. Nawaz , “Molecular Mechanism of VEGF and Its Role in Pathological Angiogenesis,” Journal of Cellular Biochemistry 123, no. 12 (2022): 1938–1965, 10.1002/jcb.30344. 36288574

[56] D. I. R. Holmes and I. Zachary , “The Vascular Endothelial Growth Factor (VEGF) Family: Angiogenic Factors in Health and Disease,” Genome Biology 6, no. 2 (2005): 209, 10.1186/gb-2005-6-2-209. 15693956 PMC551528

[57] A. L. White and G. J. Bix , “VEGFA Isoforms as Pro‐Angiogenic Therapeutics for Cerebrovascular Diseases,” Biomolecules 13, no. 4 (2023): 702, 10.3390/biom13040702. 37189449 PMC10136305

[58] J. A. Nagy , A. M. Dvorak , and H. F. Dvorak , “VEGF‐A and the Induction of Pathological Angiogenesis,” Annual Review of Pathology: Mechanisms of Disease 2 (2007): 251–275, 10.1146/annurev.pathol.2.010506.134925. 18039100

[59] J. Künnapuu , H. Bokharaie , and M. Jeltsch , “Proteolytic Cleavages in the VEGF Family: Generating Diversity Among Angiogenic VEGFs, Essential for the Activation of Lymphangiogenic VEGFs,” Biology 10, no. 2 (2021): 167. 33672235 10.3390/biology10020167 PMC7926383

[60] B. Gu , T. Yang , X. Liu , and H. Shen , “Transcriptomic Analysis of the Onchidium Reevesii Central Nervous System in Response to Cadmium,” Frontiers in Marine Science 6 (2019): 547, 10.3389/fmars.2019.00547.

[61] R. B. Heredia , S. Dueñas , and L. Castillo , “Autofluorescence as a Tool to Study Mucus Secretion in Eisenia Foetida,” Comparative Biochemistry and Physiology Part A: Molecular & Integrative Physiology 151, no. 3 (2008): 407–414, 10.1016/j.cbpa.2007.01.726. 17442604

[62] A. V. Nguyen and A. M. Soulika , “The Dynamics of the Skin's Immune System,” International Journal of Molecular Sciences 20, no. 8 (2019): 1811, 10.3390/ijms20081811. 31013709 PMC6515324

[63] S. Giokas , P. Pafilis , and E. Valakos , “Ecological and Physiological Adaptations of the Land Snail Albinariacaerulea (Pulmonata: Clausiliidae),” Journal of Molluscan Studies 71, no. 1 (2005): 15–23, 10.1093/mollus/eyi001.

[64] K. C. Ghose , “The Alimentary System of Achatina Fulica,” Transactions of the American Microscopical Society 82, no. 2 (1963): 149–167, 10.2307/3223991.

[65] U. Zylstra , “Histochemistry and Ultrastructure of the Epidermis and the Subepidermal Gland Cells of the Freshwater Snails Lymnaea Stagnalis and Biomphalaria Pfeifferi,” Zeitschrift Fur Zellforschung Und Mikroskopische Anatomie 130, no. 1 (1972): 93–134. 4340212 10.1007/BF00306996

[66] B. Plesch , “An Ultrastructural Study of the Musculature of the Pond Snail Lymnaea Stagnalis (l.),” Cell and Tissue Research 180, no. 3 (1977): 317–340, 10.1007/BF00227599. 872199

[67] A. Healey , A. Furtado , T. Cooper , and R. J. Henry , “Protocol: A Simple Method for Extracting Next‐Generation Sequencing Quality Genomic DNA from Recalcitrant Plant Species,” Plant Methods 10, no. 1 (2014): 21, 10.1186/1746-4811-10-21. 25053969 PMC4105509

[68] G. Marçais and C. Kingsford , “A Fast, Lock‐Free Approach for Efficient Parallel Counting of Occurrences of k‐Mers,” Bioinformatics (Oxford, England) 27, no. 6 (2011): 764–770. 21217122 10.1093/bioinformatics/btr011 PMC3051319

[69] H. Cheng , G. T. Concepcion , X. Feng , H. Zhang , and H. Li , “Haplotype‐Resolved De Novo Assembly Using Phased Assembly Graphs with Hifiasm,” Nature Methods 18, no. 2 (2021): 170–175, 10.1038/s41592-020-01056-5. 33526886 PMC7961889

[70] S. Koren , B. P. Walenz , K. Berlin , J. R. Miller , N. H. Bergman , and A. M. Phillippy , “Canu: Scalable and Accurate Long‐Read Assembly via Adaptive k‐mer Weighting and Repeat Separation,” Genome Research 27, no. 5 (2017): 722–736, 10.1101/gr.215087.116. 28298431 PMC5411767

[71] G. Benson , “Tandem Repeats Finder: A Program to Analyze DNA Sequences,” Nucleic Acids Research 27, no. 2 (1999): 573–580, 10.1093/nar/27.2.573. 9862982 PMC148217

[72] Z. Xu and H. Wang , “LTR_FINDER: An Efficient Tool for the Prediction of Full‐Length LTR Retrotransposons,” Nucleic Acids Research 35 (2007): W265–W268, 10.1093/nar/gkm286. 17485477 PMC1933203

[73] D. Ellinghaus , S. Kurtz , and U. Willhoeft , “LTRharvest, an Efficient and Flexible Software for De Novo Detection of LTR Retrotransposons,” BMC Bioinformatics [Electronic Resource] 9 (2008): 18, 10.1186/1471-2105-9-18. 18194517 PMC2253517

[74] S. Ou and N. Jiang , “LTR_retriever: A Highly Accurate and Sensitive Program for Identification of Long Terminal Repeat Retrotransposons,” Plant Physiology 176, no. 2 (2018): 1410–1422, 10.1104/pp.17.01310. 29233850 PMC5813529

[75] M. Tarailo‐Graovac and N. Chen , “Using RepeatMasker to Identify Repetitive Elements in Genomic Sequences,” Current Protocols in Bioinformatics 25, no. 4 (2009): 4.10.1–4.10.14, 10.1002/0471250953.bi0410s25. 19274634

[76] K. Lagesen , P. Hallin , E. A. Rødland , H.‐H. Stærfeldt , T. Rognes , and D. W. Ussery , “RNAmmer: Consistent and Rapid Annotation of Ribosomal RNA Genes,” Nucleic Acids Research 35, no. 9 (2007): 3100–3108, 10.1093/nar/gkm160. 17452365 PMC1888812

[77] P. P. Chan and T. M. Lowe , “tRNAscan‐SE: Searching for tRNA Genes in Genomic Sequences,” Methods in Molecular Biology 1962 (2019): 1–14. 31020551 10.1007/978-1-4939-9173-0_1 PMC6768409

[78] E. P. Nawrocki and S. R. Eddy , “Infernal 1.1: 100‐fold Faster RNA Homology Searches,” Bioinformatics 29, no. 22 (2013): 2933–2935, 10.1093/bioinformatics/btt509. 24008419 PMC3810854

[79] C. Holt and M. Yandell , “MAKER2: An Annotation Pipeline and Genome‐Database Management Tool for Second‐Generation Genome Projects,” BMC Bioinformatics 12 (2011): 491, 10.1186/1471-2105-12-491. 22192575 PMC3280279

[80] I. Korf , “Gene Finding in Novel Genomes,” BMC Bioinformatics 5 (2004): 59, 10.1186/1471-2105-5-59. 15144565 PMC421630

[81] B. J. Haas , S. L. Salzberg , and W. Zhu , “Automated Eukaryotic Gene Structure Annotation Using EVidenceModeler and the Program to Assemble Spliced Alignments,” Genome Biology 9, no. 1 (2008): R7, 10.1186/gb-2008-9-1-r7. 18190707 PMC2395244

[82] Z. Yang , “PAML 4: Phylogenetic Analysis by Maximum Likelihood,” Molecular Biology and Evolution 24, no. 8 (2007): 1586–1591, 10.1093/molbev/msm088. 17483113

[83] B. Langmead and S. L. Salzberg , “Fast Gapped‐Read Alignment with Bowtie 2,” Nature Methods 9, no. 4 (2012): 357–359, 10.1038/nmeth.1923. 22388286 PMC3322381

[84] M. I. Love , W. Huber , and S. Anders , “Moderated Estimation of Fold Change and Dispersion for RNA‐seq Data with DESeq2,” Genome Biology 15, no. 12 (2014): 550, 10.1186/s13059-014-0550-8. 25516281 PMC4302049

[85] T. Wu , E. Hu , S. Xu , et al., “clusterProfiler 4.0: A Universal Enrichment Tool for Interpreting Omics Data,” Innovation 2, no. 3 (2021): 100141. 34557778 10.1016/j.xinn.2021.100141 PMC8454663

[86] K. J. Livak and T. D. Schmittgen , “Analysis of Relative Gene Expression Data Using Real‐Time Quantitative PCR and the 2−ΔΔCT Method,” Methods 25, no. 4 (2001): 402–408, 10.1006/meth.2001.1262. 11846609

