# Supplementary material for: T2T Genome Assembly and Multi‐Omics Data Reveal Terrestrial Adaptation and Mucus Biosynthesis in Tropical Leatherleaf Slug (Laevicaulis alte)
Source: Adv Sci (Weinh). 2026 Jun 15:e76129. Online ahead of print. doi: 10.1002/advs.76129 (PMC13336835; doi:10.1002/advs.76129)
Supplement: Supplementary file 1 — Supporting File 1: advs76129‐sup‐0001‐SuppMat.pdf. [file ADVS-9999-e76129-s002.pdf]

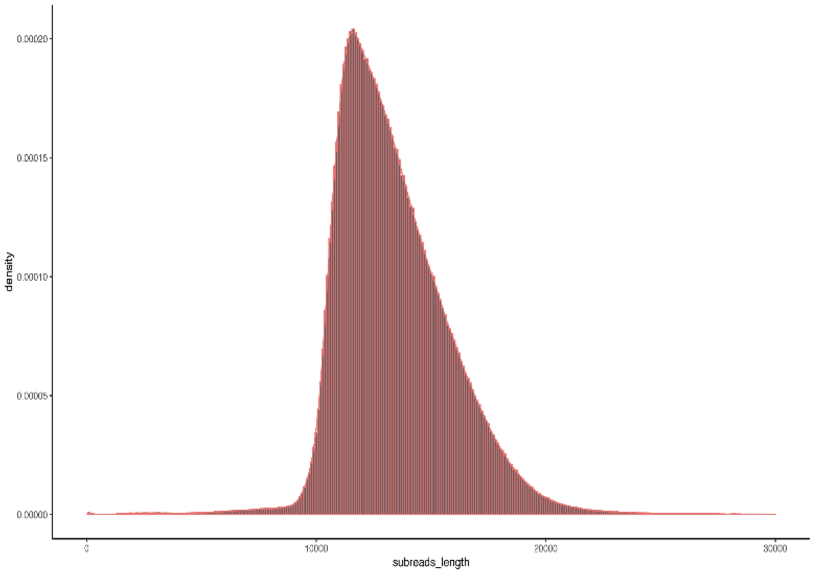

Fig. S1 The PacBio HiFi length distribution.

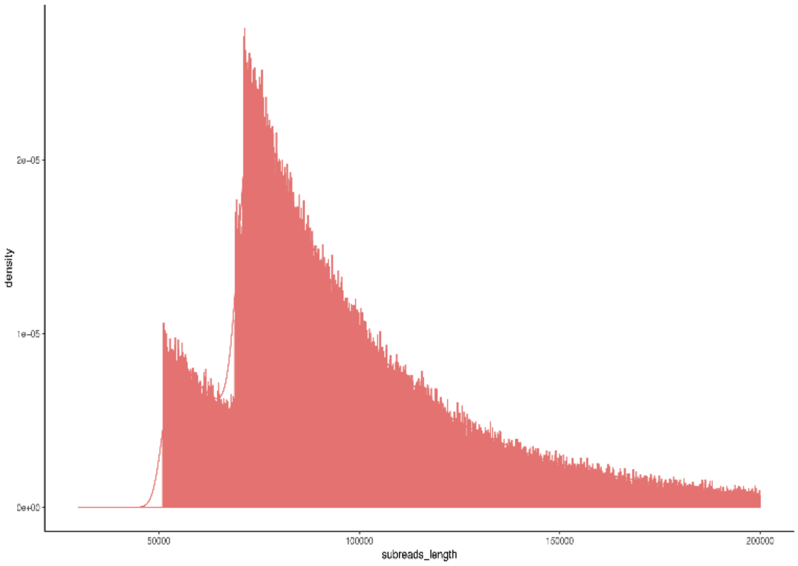

Fig. S2 The Nanopore ONT length distribution.

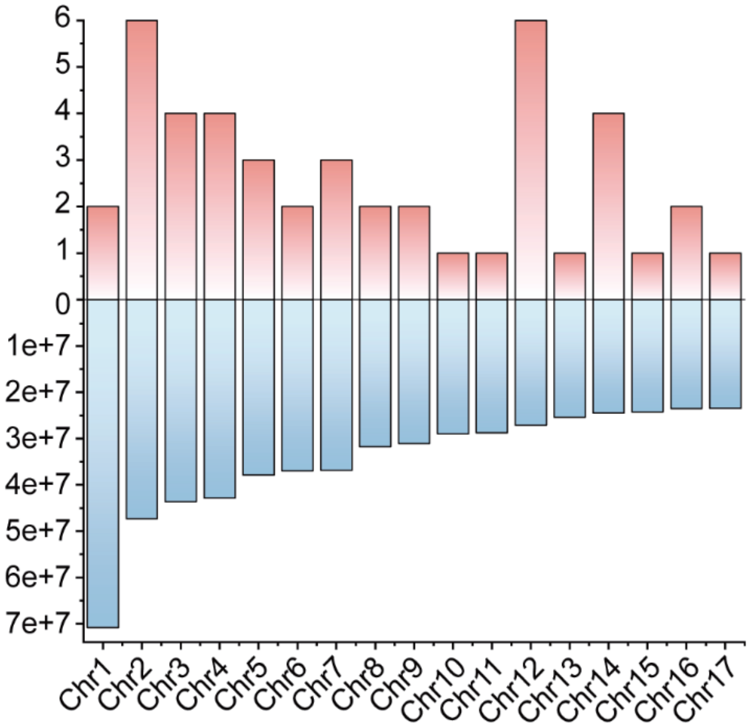

Fig. S3 The chromosome length vs contig counts.

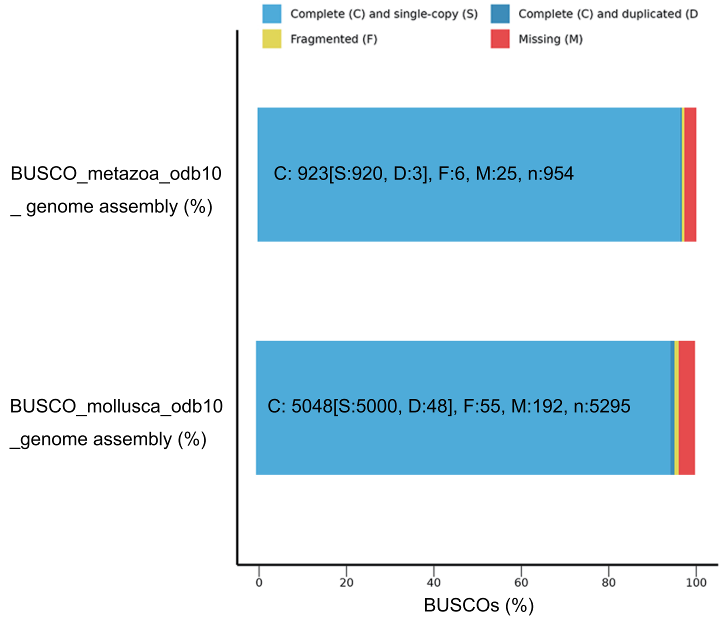

Fig. S4 BUSCO evaluation of *L. alte* genome integrity

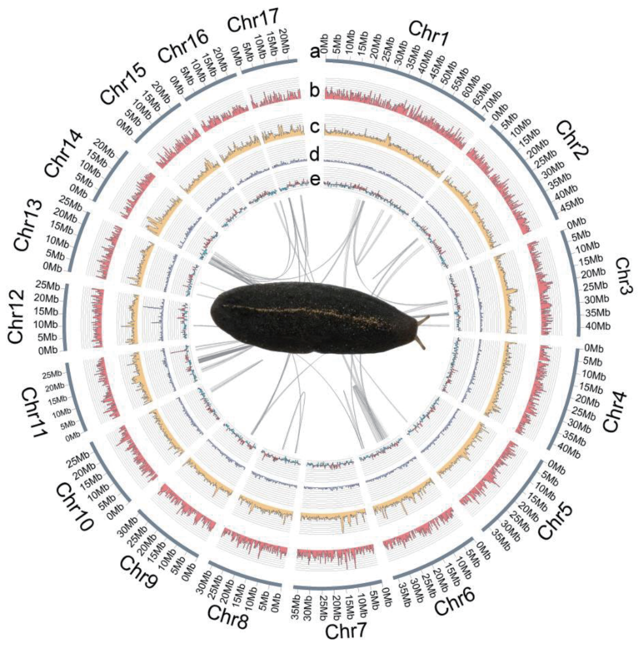

Fig. S5 The Circos plot of genomic features and synteny provides a layered view of
. genomic features

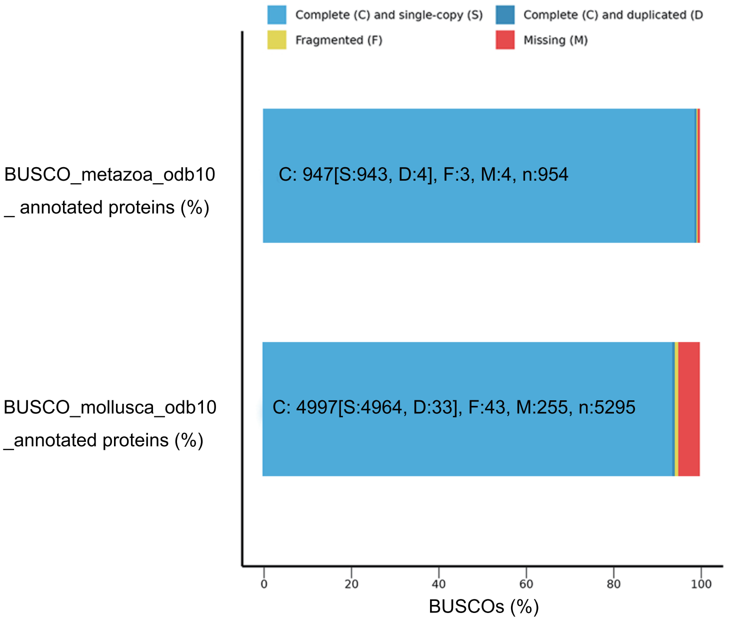

Fig. S6 BUSCO evaluation of *L. alte* genome structure annotation

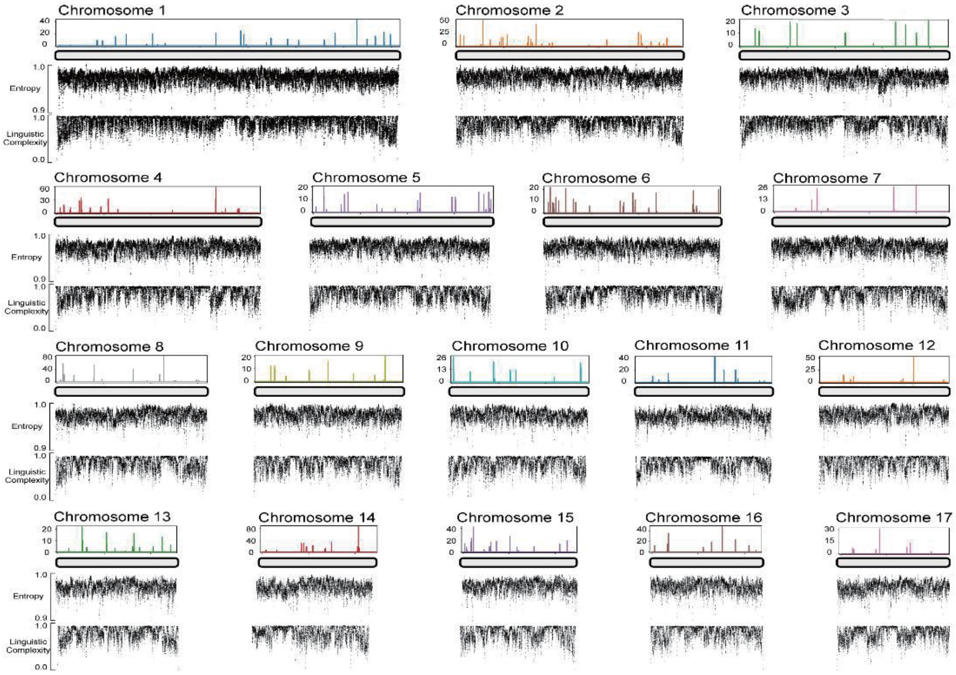

Fig. S7 The entropy plots for all 17 chromosomes of *L. alte*. The unordered scaffolds within a chromosome are shaded alternately white and gray. The entropy plots indicate that most of these 17 chromosomes maintain a relatively high baseline of compositional diversity, punctuated by localized pockets of lower complexity.

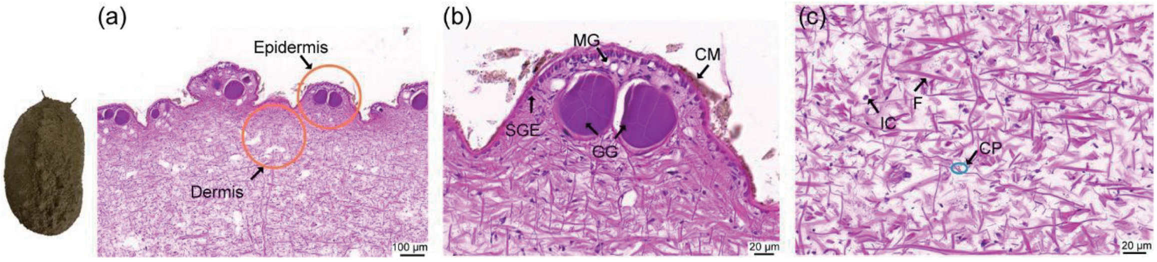

Fig. S8 H&E-stained sections of dorsal skin tissue from *Onchidium reevesii*. (a) 10×; (b) 40×, The epidermis in the dorsal skin of *O. reeveii*; (c) 40×, The dermis in the dorsal skin of *O. reeveii*; MG, mucous gland; CM, cuticular membrane; SGE, stratum germinative; GG, granular gland; F, fibers; IC, immune cell; CP, calcium particle.

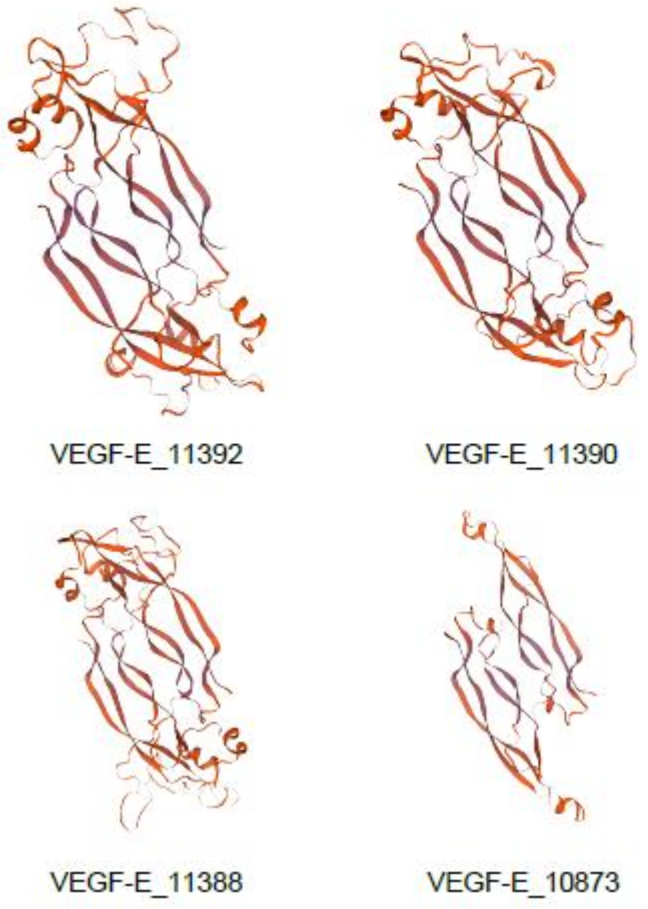

Fig. S9 The 3D structure models of VEGF-E_10873, VEGF-E_11388, VEGF-E_11390, and VEGF-E_11392
